# Chirality in the Solid State: Chiral Crystal Structures in Chiral and Achiral Space Groups

**DOI:** 10.3390/ma15175812

**Published:** 2022-08-23

**Authors:** Gerhard H. Fecher, Jürgen Kübler, Claudia Felser

**Affiliations:** 1 Max Planck Institute for Chemical Physics of Solids, D-01187 Dresden, Germany; 2 Institute of Solid State Physics, Technical University Darmstadt, D-64289 Darmstadt, Germany

**Keywords:** chirality, chirality measure, chiral space groups, chiral elements, compounds, and surfaces, electronic structure, dichroism

## Abstract

Chirality depends on particular symmetries. For crystal structures it describes the absence of mirror planes and inversion centers, and in addition to translations, only rotations are allowed as symmetry elements. However, chiral space groups have additional restrictions on the allowed screw rotations as a symmetry element, because they always appear in enantiomorphous pairs. This study classifies and distinguishes the chiral structures and space groups. Chirality is quantified using Hausdorff distances and continuous chirality measures and selected crystal structures are reported. Chirality is discussed for bulk solids and their surfaces. Moreover, the band structure, and thus, the density of states, is found to be affected by the same crystal parameters as chirality. However, it is independent of handedness. The Berry curvature, as a topological measure of the electronic structure, depends on the handedness but is not proof of chirality because it responds to the inversion of a structure. For molecules, optical circular dichroism is one of the most important measures for chirality. Thus, it is proposed in this study that the circular dichroism in the angular distribution of photoelectrons in high symmetry configurations can be used to distinguish the handedness of chiral solids and their surfaces.

## 1. Introduction

Compounds without a center of inversion, that is, non-centrosymmetric compounds, are of particular interest because of their symmetry-dependent physical properties in addition to hosting various interesting topological properties. Pyroelectricity, ferroelectricity, piezoelectricity, and optical activity or nonlinear optical behavior are employed in numerous applications. These properties require the absence of centrosymmetry. However, describing a compound simply as non-centrosymmetric is insufficient because its properties depend on additional details of the crystalline structure. Consequently, various relations between crystallographic structures, symmetries, and physical properties of non-centrosymmetric compounds were proposed and are summarized in Table 1 (compare Ref. [1]). Second-order nonlinear optical behavior (second harmonic generation) possesses symmetry requirements similar to that of piezoelectricity. Cubic compounds are either chiral (Laue classes 23, 432) or solely piezoelectric (Laue class $\bar{4}3m$). Gyrotropic crystals [2] are only found among the groups in the first three classes of Table 1. The gyrotropic point group symmetry cannot differentiate between axial and polar vectors, and only structures from the first non-centrosymmetric class (Laue classes 1,…,4, and 6) are both enantiomorphous and polar.

In this study, chiral structures were focused upon. The definition of chirality was presented in 1894 by Lord Kelvin: “*I call any geometrical figure, or group of points, chiral, and say that it has chirality, if its image in a plane mirror, ideally realized, cannot be brought to coincide with itself*.” [3,4]. For an object to be chiral, it must not possess either a mirror plane, a center of inversion, or any rotation–reflection axes [5]. If an object possesses either one of these symmetry elements, it can be superimposed on its mirror image and is, therefore, achiral [3,5]. However, when considering terms such as chirality and handedness further attention is needed [6]. (The terms chirality and handedness are often used in very different contexts. Cintas traced their use in chemistry in a comprehensive essay [7].) As mentioned above, chirality is defined by the lack of certain features of symmetry, thereby resulting in an object that cannot be superimposed on its mirror image. Handedness classifies (chiral) objects into right-handed and left-handed [8]. Handed geometric objects are chiral, but not all chiral objects are handed [9], and this is referred to as Ruch’s shoe–potato problem [6,9].

**Figure 1:**
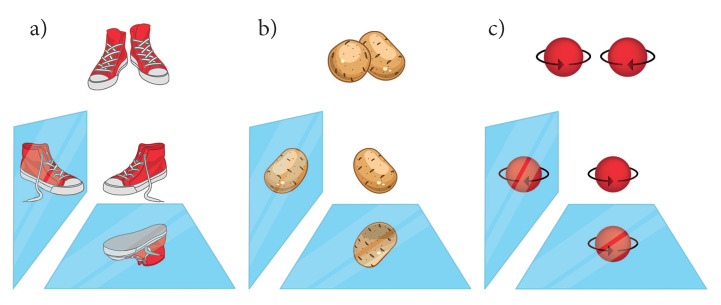
Chirality and handedness. (**a**) A pair of shoes is chiral and handed. The right shoe is the mirror image of the left shoe, but none of the mirror images coincide with itself; (**b**) potatoes are chiral because there exists no mirror operation that transform them into themselves; however, they are not handed, and there exist no left- or right-handed potatoes; (**c**) a rotating ball can exhibit a left (clockwise) or a right (*“handed”*) (anti-clockwise) rotation and thus it may be called handed; however, one of its mirror images coincided with itself and therefore it is not chiral but helical.

Moreover, handedness is sometimes also assigned to the helicity (rotational direction) (In chemistry, helicity assigns the rotation sense of screw-type entities e.g.: a helix in molecules [5]. In physics, it stands also for the projection of the spin on the linear momentum) of rotating objects. The rotation is a result of a cross-product. In addition, it is invariant under horizontal mirror operations and thus achiral. In general, right- and left-(handed) rotations are not necessarily chiral although they are characterized considering handedness. The situations are illustrated in Figure 1.

It is worthwhile to note that there exist several more definitions of chirality and handedness in other disciplines, for instance in magnetism, particle physics, cosmology, or biology. As an example, optical handedness is defined in Ref. [4] as: *“A chiral medium is called optically right- or left-handed according as the propagational velocity of right-handed or of left-handed circularly polarized light travelling through it is the greater”*. The most part of the present work is related to the chirality of the crystal structure which is a static problem, in contrast to the chirality of elementary particles which is a dynamic problem. L.D. Barron [10] distinguished these two cases by the terms true or false chirality, respectively. The definition of static chirality by Barron is: *“true (static) chirality is exhibited by systems that exist in two distinct enantiomeric states that are interconverted by space inversion (parity), but not by motion reversal (time reversal), combined with any proper spatial rotation”* [11]. K. Mislow [12] criticized the true/false characterization of Barron as infelicitous and used the terms geometric and motion-dependent chirality instead. The dynamical aspect of chirality plays a major role in magnetic systems, as was reviewed by Togawa et al. [13], Cheong and Xu [14], and Inoue [15]. Chiral molecular magnets were reviewed by Train et al. [16]. However, the present work concentrated on crystalline systems with non-magnetic space groups.

In the following, tables are presented that provide the necessary information for the classification of chiral compounds in terms of space group symmetry and their surfaces by plane groups. Subsequently, various examples are presented, and, furthermore, a set of chiral compounds, which was of particular interest, is selected. Their crystal structures are listed and for certain selected materials the electronic band structures are reported. Finally, the relation between chirality and circular dichroism via photoelectron spectroscopy is demonstrated for two selected compounds.

## 2. Chirality in Three Dimensions

The symmetry of the three-dimensional solids can be described using space groups. (the hierarchy of the crystallographic space groups is explained for example in Ref. [17]). When considering crystals, the definition of chirality is [18]: *A crystal is to be considered chiral if its space group contains only proper operations*. To determine the chiral crystals, only those crystals that have space groups satisfying this definition must be identified. The overall 230 space groups can be divided into three classes. Class I comprises the 165 space groups that contain at least one improper operation (inversion, mirror, glide, or $S_{n}$ (a rotation followed by a reflection in a plane perpendicular to the axis of rotation) operations). In addition, solids in these space groups are always achiral even if the 3-dimensional asymmetric unit is chiral. Class II comprises the 22 chiral space groups (11 enantiomorphous pairs) that contain at least one screw axis that is not the $2_{1}$-screw axis. Solids in these space groups are always chiral even in cases where the asymmetric unit is achiral. Finally, class III is often considered confusing, where it comprises the 43 space groups that contain only proper rotations and the $2_{1}$-screw rotation. Although there are no reflections and inversions, among other things, these space groups are achiral. Moreover, although Class III space groups are achiral, the crystals that are fabricated using them always have a chiral structure. Table 2 summarizes the 65 space groups under classes II and III, wherein the chiral crystal structures are determined. These space groups are referred to as the Sohncke groups. The subset of chiral Sohncke groups is asymmorphic.

As mentioned above, not all of those 65 space groups are chiral when considered individually, although the crystal structures in these space groups are always chiral. The 43 class III groups are transformed into themselves when the atomic positions are inverted, thereby demonstrating that they are achiral. Moreover, the inversion of the atomic positions results in a reversal of the chirality sense in the accompanied chiral structures or in at least one of their substructures.

**Figure 2:**
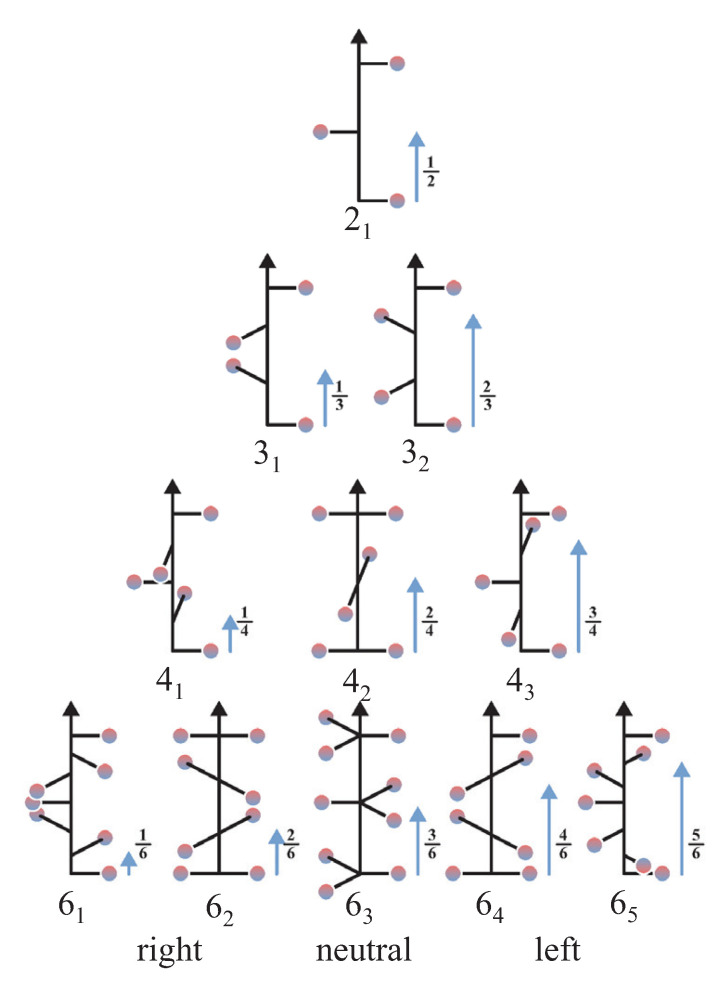
The 2-, 3-, 4-, and 6-fold screw axes. For each screw, the relative translation $\tau =\frac{n}{N}$ along the *z* axis after the rotation is assigned by an arrow. For the neutral axes a positive or negative rotation about the *z*-axis (e.g., rotation by ±π in case of the $2_{1}$ screw rotation) yields the same result.

The chiral crystal structures are related to screw axes. The various 2-, 3-, 4-, and 6- fold screw rotations are shown in Figure 2. Via inversion, screw rotations $N_{n}$ are transformed into a screw rotation $N_{m}$, where m+n=N. $N_{n}$ implies a rotation by 2π/N followed by a shift of τ=n/N applied to a lattice point, where τ is in relative coordinates. Consequently, the following pairs of enantiomorphous screw rotations exist: $\left\{3_{1},3_{2}\right\}$, $\left\{4_{1},4_{3}\right\}$, $\left\{6_{1},6_{5}\right\}$, and $\left\{6_{2},6_{4}\right\}$, with each being chiral. The three screw rotations $2_{1}$, $4_{2}$, and $6_{3}$ are invariant under inversion and thus cannot be considered as enantiomorphous pairs. These neutral screw rotations with n=N/2 are achiral, that is, they have no enantiomorphous partner, although an atomic distribution around any of these screw axes is chiral. Moreover, the neutral screws do not have a definite helicity. Further, the pure screws $N_{n}$ are those where *N* and *n* have no common divisor ($2_{1}$, $\left\{3_{1},3_{2}\right\}$, $\left\{4_{1},4_{3}\right\}$, $\left\{6_{1},6_{5}\right\}$). $2_{1}$ is the only neutral as well as pure screw [19]. $4_{2}$ and $\left\{6_{2},6_{4}\right\}$ result from combinations of $2_{1}$ or $\left\{3_{1},3_{2}\right\}$ screws with a 2-fold rotation axis, whereas $6_{3}$ screws imply a 3-fold rotational symmetry. Regarding the screw axes that are not neutral, n<N/2 results in right and n>N/2 in left screws (see Figure 2). In general, the screw axes for n>N/2 may also be realized with negative values $n^{\prime }=n-1$, where, for example, $3_{2}\equiv 3_{-1}$.

The $\left\{3_{1},3_{2}\right\}$, $\left\{4_{1},4_{3}\right\}$, $\left\{6_{1},6_{5}\right\}$, and $\left\{6_{2},6_{4}\right\}$ screw rotations result in the appearance of the 22 chiral groups of class II. The first partner of these pairs is defined as right-handed while the second as left-handed. The 22 chiral-helical space groups that occur as 11 enantiomorphous pairs are tabulated in Table 3. Most of the databases for crystal structures typically provide only one of the chiral space groups for the chiral structures because the second one is redundant. For example, in the Pearson database [20] only structures with space group number 213 can be found but not with number 212.

Within the cubic space groups only one enantiomorphous pair exists, which comprises the groups $P\;4_{3}32$ (212) and $P\;4_{1}32$ (213). The hexagonal crystal systems contain four pairs, whereas the trigonal or tetragonal systems comprise three pairs each. Further, orthorhombic or lower symmetry crystal systems do not contain enantiomorphous pairs and thus the related space groups are achiral. Thus, only primitive (*P*) space groups out of the Sohncke groups are chiral, whereas the centered ones are not (*C*, *I*, or *F*).

Two types of symmetry axes, chiral and polar, can be defined to describe the properties of the Sohncke groups. According to Ref. [1], a direction is referred to as polar if its two directional senses are geometrically or physically different, and a polar symmetry direction of a crystal is referred to as a polar axis. Only proper rotation or screw axes can be polar. Moreover, as mentioned earlier, chiral and polar are not the same things. Polar axes are linked to a polar property or geometry whereas chiral axes are linked to a chiral property such as enantiomorphism. In Ref. [21], the chiral axes were defined in analogy to polar axes. Table 4 lists the chiral and polar axes of the Sohncke groups, and the ones of the remaining non-centrosymmetric structures can be found in Refs. [1,21] along with all nonchiral and non-polar directions. The symmetry axes of different types coincide only for the Laue classes 2, 3, 4, and 6, whereas class 1 has no definite symmetry axes.

## 3. Chirality in Two Dimensions

Chirality in reduced dimensions is crucial to solid surfaces or projections of the crystal structure along a particular direction. In such cases, the symmetry is described by the 17 plane space groups or wallpaper groups. Out of the 17 groups, only 5 contain pure rotations, namely, the groups pn with n=1, 2, 3, 4, and 6 which contain the purely rotational subgroups $C_{n}$ of the full rotational group SO(3). The remaining 12 groups describe achiral objects.

The plane space groups are listed in Table 5 (compare Refs. [22,23]) coupled with those marked that describe chiral objects. These five groups are often referred to as the chiral plane groups, which is somewhat misleading because they do not exist in enantiomorphous pairs and thus, are not chiral by themselves. However, objects that adopt their symmetry are chiral similar to the achiral subset of the Sohncke groups in three dimensions.

## 4. Chirality Measures

Determining and quantifying the chirality and handedness of molecular and crystalline structures can be challenging. Problems related to the crystallography of chiral compounds and their structure were discussed in Refs. [24,25], whereas those related to quantifying chirality were discussed vividly in [6,26,27,28,29,30,31,32]. Chirality measures are special cases of symmetry measures [33]. Various measures for chirality have been proposed in the literature that are mostly based on distances [34,35]. Avnir’s continuous chirality measures are based on the mean square of distances [36,37,38,39]. Although the chirality measures have in most cases been developed for chiral molecules, they may be extended to solids through certain modifications [40].

The Hausdorff distances [34,41,42] are defined by the Euclidian distances $d(\hat{x},{\hat{x}}^{\prime })$ between the points $\hat{x}$ of a structure *X* and those (${\hat{x}}^{\prime }$) of a reference structure $X_{\mathrm{ref}}$ (here: sup:= supremum, that is the least upper bound and inf:= infimum, that is the greatest lower bound):

(1) $$\begin{array}{ccc} h(q) & = & \underset{\hat{x}\in X}{\sup}g\left(\hat{x}\left(q\right)\right),\\ g(\hat{x}\left(q\right)) & = & \underset{{\hat{x}}^{\prime }\in X_{\mathrm{ref}}}{\inf}d\left(\hat{x}\left(q\right),{\hat{x}}^{\prime }\right). \end{array}$$

for a particular parameter *q* or a set of parameters depending on the dimensionality. The abstract parameter set *q* will later be identified as the position parameters of the atoms in crystals. To determine a chirality measure, the reference system is usually chosen to be achiral. h(q) is always positive by definition, because $d\left(x,x^{\prime }\right)=\sqrt{{\left(x-x^{\prime }\right)}^{2}}$. Further, the Hausdorff distance may be normalized to $H\left(q\right)=h\left(q\right)/h_{\max}$ or similar by choosing a suitable value and reference system for $h_{\max}$. However, herein, primarily unnormalized Hausdorff distances were used.

The unnormalized (lower case) and normalized (upper case) continuous chirality measures are defined by:

(2) $$\begin{array}{ccc} s^{2}\left(G\right) & = & \sum_{i=1}^{n}{∥p_{i}-p_{i}^{\mathrm{sym}}∥}^{2},\\ S^{2}\left(G\right) & = & \frac{s^{2}\left(G\right)}{N}\\ N & = & \sum_{i=1}^{n}{∥p_{i}^{\mathrm{chiral}}-p_{i}^{\mathrm{sym}}∥}^{2}. \end{array}$$

where $∥\ldots ∥$ is the norm and *G* assigns an abstract manifold or group of parameters, that is related to the appearance of the investigated system. For example, it may comprise the symmetry operations and structural parameters of molecules or crystals. Here, $p_{i}$ are the points in the actual crystal structure depending on the positions of the atoms, $p_{i}^{\mathrm{sym}}$ are the closest points of the nearest achiral structure, and $p_{i}^{\mathrm{chiral}}$ are the points of the chiral structure where *N* is at maximum. Alternatively, the square root of the continuous chirality measure: $S\left(G\right)=\sqrt{S^{2}\left(G\right)}$ may be used. When normalized, it has the same range 0≤S≤1 as its square $S^{2}$. A square root normalization was also used in the chirality functions of Cossé-Barbi and Raji [43].

Apart from $S^{2}\left(G\right)$, the chirality measure S(G) can be directly interpreted as a type of distance similar to the Hausdorff measure; however, it exhibits different behavior. Hausdorff distance and continuous chirality measure are of different characters. h(q) is an extremal and $S^{2}\left(G\right)$ an average property (mean square deviation). In cubic systems, relative position parameters are used, such that both measures, continuous chirality and Hausdorff, are independent of the lattice parameter *a*. In contrast, in tetragonal or hexagonal systems the c/a ratio must be respected as well if the *z*-parameter is provided relative to *c*.

An achiral structure may be determined by averaging over the positions of an enantiomorphous pair of structures. In addition, the chirality measures may be defined in slightly different ways as well. In principle, the distances between the positions of the enantiomorphous pair of structures may also be employed as a measure of chirality.

**Figure 3:**
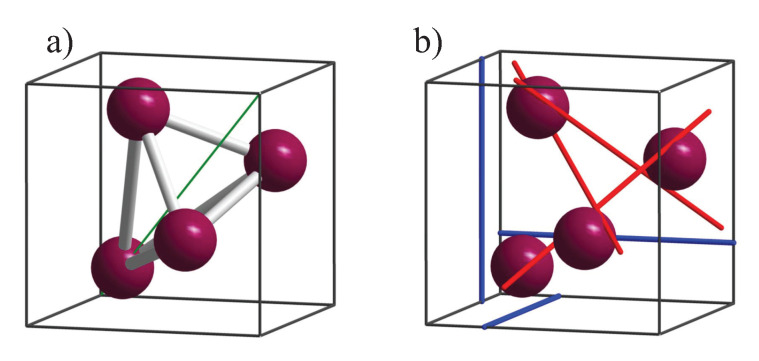
Screw rotations and simple structure in space group 198. (**a**) Arrangement of the atoms on position 4a. (**b**) Position of the 2- (blue) and 3-fold (red) screw axes. The three $2_{1}$ screw axes are parallel to the principle axes. The three $3_{2}$ screw axes are parallel to $\langle \bar{1}11\rangle$ type axes. The [111] axis shown in a) (green diagonal line) is a simple 3-fold rotational axis.

The chirality measures are illustrated by means of a simple cubic structure with four atoms in the primitive cell, where the basis is the space group $P\;2_{1}3$ with one Wyckoff position 4a (u,u,u) occupied, as illustrated in Figure 3a ((u,u,u) is used for the position to avoid confusion with the coordinates x,y,z). For special values of *u* (e.g., ±18), the symmetry changes to one of the enantiomorphous space groups $P\;4_{3}32$ or $P\;4_{1}32$.

Space group $P\;2_{1}3$ contains 12 symmetry elements. In addition to the unity operator, these are three 2-fold screw rotations, five simple, 3-fold rotations, and three 3-fold screw rotations. The three axes for the $2_{1}$ screw rotations are parallel to the principle axes along the lines [14,0,*z*], [0,*y*,14], and [*x*,14,0]. For instance, one of the axis of a simple $C_{3}$ rotation is along [1,1,1] (for others see [22]). Further, the three $3_{2}$ screw rotations have axes parallel to [$\bar{1}$,1,1], [1,1,$\bar{1}$], [1,$\bar{1}$,1] that is in detail along the lines [*x* + 16,$\bar{x}$ + 16,$\bar{x}$], [$\bar{x}$ + 13,$\bar{x}$ + 16,*x*], and [$\bar{x}$−16,*x* + 13,$\bar{x}$]. The axes of the six screw rotations are illustrated in Figure 3b. These six screw rotations are the same in the enantiomorphous space groups 212 and 213, which contain in addition three more $2_{1}$ as well as overall six 4-fold ($4_{1}$ and $4_{3}$) screw rotations as symmetry elements.

As an achiral reference system for the determination of the chirality measures, a face-centered cubic lattice (fcc) was used. The positions of the atoms in the three types of structures are compared in Table 6. It is evident that the chiral simple cubic structures are produced by shifting the atoms out of the high symmetry positions of the achiral fcc structure with space group $F\;m\bar{3}m$.

**Figure 4:**
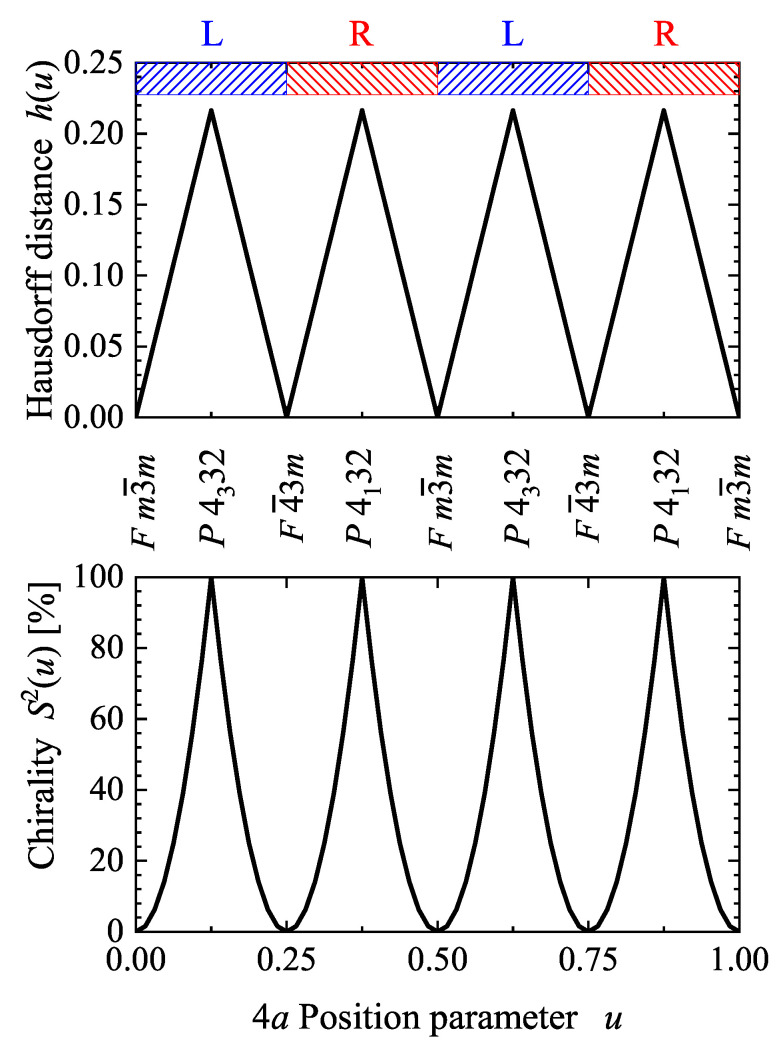
Hausdorff distance h(u) and continuous chirality measure $S^{2}\left(u\right)$ in a chiral cubic compound based on space group 198 with a single 4a Wyckoff position occupied. Shown are the dependencies of h(u) and $S^{2}\left(u\right)$ on the position parameter *u* of the Wyckoff position 4a. The character (R and L) of structures in space group 198 is adopted from the handedness of the closest chiral space group 213 or 212.

Figure 4 shows the Hausdorff distance h(u) and continuous chirality measure $S^{2}\left(u\right)$ with variation of the *u* parameter of the 4a Wyckoff position in space group 198, $P\;2_{1}3$. For special values of *u*, the structure adopts a higher symmetry. Both, H(u) and $S^{2}\left(u\right)$, vanish for u=i/4,i∈N when the structure adopts the Cu or A1 type [44] fcc structure with space group $F\;m\bar{3}m$. The chirality is largest for u=1/8, 6/8 or u=3/8, 7/8 where a structure with one of the chiral space groups $P\;4_{3}32$ or $P\;4_{1}32$ was adopted. The latter two cases may be referred to as ideal chiral structures. The $2_{1}$ screw axis of $P\;2_{1}3$ does not have a definite helicity; therefore, the handedness (R for right and L for left) for variation of *u* may be adopted from the closest chiral space group with $4_{3}$ or $4_{1}$ screw axes. Space group 198 is a subgroup of the enantiomorphous groups 212 or 213, accordingly, structures or substructures that are close to one of these are assigned as an L or R type structure.

## 5. Chiral Systems: From Crystalline Elements to Compounds

### 5.1. Elements with Chiral Structures

The only elemental metal with a chiral structure is β-Mn (Mn, cP20, 213, $P\;4_{1}32$) [20] (here and subsequently, the prototypes are provided in braces by name, Pearson symbol, number, and name of the space group according to [20] and following the Nomenclature of Inorganic Chemistry – IUPAC recommendations 2005 [45].). The Strukturbericht notation for this structure is A13 [44]. Two other chiral elements are Se and Te, with the prototype structures (Se, hP3, 152, $P\;3_{1}21$) or (Te, mP4, 4, $P\;12_{1}1$), where the latter is a high pressure phase.

Here, the A8 [44] crystal structure of γ-Se (Se, hP3, 152, $P\;3_{1}21$) is of particular interest as the space group $P\;3_{1}21$ is chiral in an enantiomorphous pair (see Table 3). The inverted structure belongs to the enantiomorphous space group $P\;3_{2}21$ (154). In the first type (152), the Wyckoff positions are 3a with (0.23, 0, 1/3), and in the second type (154), the Wyckoff positions are 3a with (0.23, 0, 2/3). Equivalently, it ispossible to change 3a from (0.77, 0.77, 0) to (0.23, 0.23, 0) while going from $P\;3_{1}21$ to $P\;3_{2}21$. A pure inversion of the atomic position in the same space group would result in an entirely different structure and not only in a different helicity because the 3a type would be changed to a 6c type position. Moving the atom from 3a with (0.23, 0, 2/3) to (1/3, 0, 2/3) changes the symmetry to space group $R\;\bar{3}m$ (166) while the structure becomes achiral. However, Se at high pressure (>80 GPa) adopts a β-Po structure, which also belongs to space group $R\;\bar{3}m$ with the atoms on (0, 0, 0) [46].

**Figure 5:**
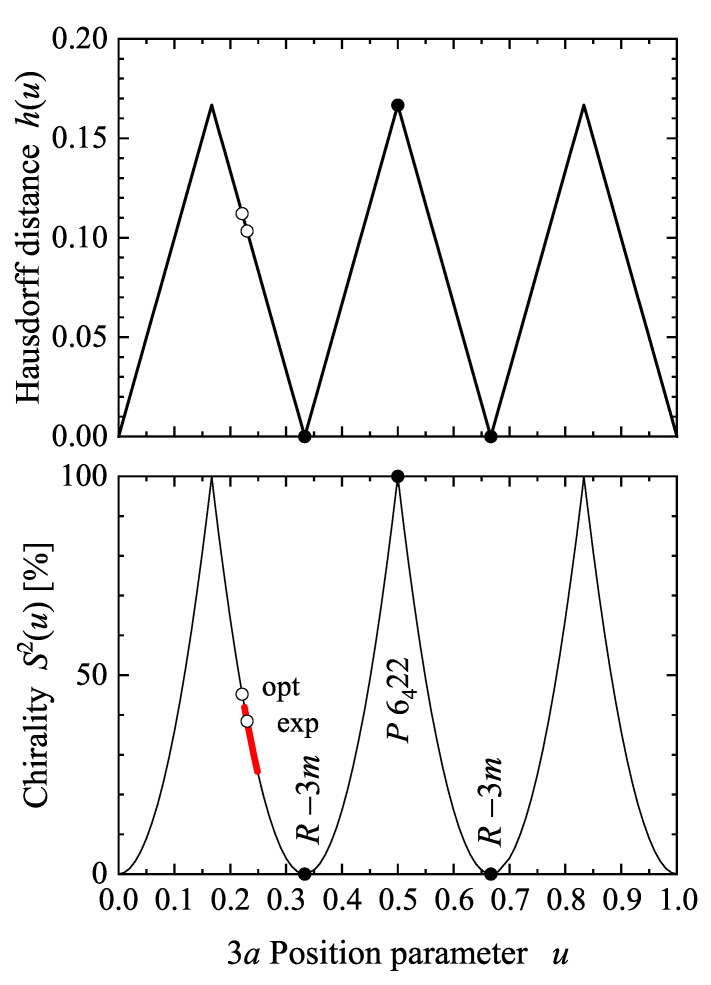
Chirality of the A8 structure of γ-Se. Shown are the Hausdorff distance *h* and the continuous chirality measure $S^{2}$ as a function of the internal parameter *u*. Open symbols assign the values at optimized and experimental *u* values (Section 7.1) and closed symbols mark the parameters where the space group changes away from $P\;3_{1}21$. The range of *u* and $S^{2}$ under pressure in the experiment [47] is marked by the thick red line.

The chirality measures *h* and $S^{2}$ of the (Se, hP3, 152, $P\;3_{1}21$) structure are shown in Figure 5. The maxima of the chirality measure appear at 1/6, 1/2, and 7/6. At u=1/2, the structure adopts the $P\;6_{2,4}22$ space group, depending on whether it started with the $3_{1}$ or $3_{2}$ screw rotation. As expected, the chirality measure becomes zero at u=0,1/3,2/3, and 1 when the structure becomes achiral. Further, at $u_{\mathrm{Se}}=0.23$ and $u_{\mathrm{Te}}=0.2636$ as reported for Se and Te, the chirality measures are approximately $S_{Se}^{2}=38.4$% and $S_{Te}^{2}=17.5$%, respectively. Moreover, the *u* parameter can be varied by external pressure. In experiments performed on Se, it varies from 0.2254 under ambient conditions to 0.2487 at a pressure of 86 GPa [47], which results in a decrease of $S_{\mathrm{Se}}^{2}$ from 41.9 to 25.8%.

### 5.2. Compounds with Chiral, Cubic Structures

This paragraph addresses cubic structures, of which certain have recently been very prominent in work on topological materials [48,49]. As mentioned above, within the cubic space groups, only one enantiomorphous pair exists, comprising $P\;4_{3}32$ (212) and $P\;4_{1}32$ (213). Moreover, these groups are not polar, implying that crystals with this symmetry do not exhibit pyroelectricity. There are two simple binary chiral compounds reported to belong to the chiral cubic space group 213, $P\;4_{1}32$, which are BaSi${}_{2}$ and SrSi${}_{2}$. The prototype structure is (SrSi${}_{2}$, cP12, 213, $P\;4_{1}32$). Other binaries crystallizing in space group 213 are more complicated and have much more atoms in the primitive cell. Certain prototype structures are (space group information not repeated) (Mg${}_{3}$Ru${}_{2}$, cP20), (V${}_{8}$C${}_{7}$, cP60), (K${}_{6}$Sn${}_{25}$, cP124), (K${}_{8}$Sn${}_{25}$, cP136), and (RuZn${}_{6}$, cP252). Certain prototypes for ternaries in this space group are (Mo${}_{3}$Al${}_{2}$C, cP24), (CsBe${}_{2}$F${}_{5}$, cP32), (Ag${}_{3}$AuS${}_{2}$, cP48), (Zn${}_{2}$Ge${}_{3}$O${}_{8}$, cP52), (Cu${}_{3}$Mn${}_{3}$O${}_{8}$, cP56), and (LiFe${}_{5}$O${}_{8}$, cP56).

Several alloys exist that crystallize in the A13 structure of β-Mn. They have non-integer and random site occupations. Ordered derivatives of this structure are the binary prototype (AlAu${}_{4}$, cP20, 198, $P\;2_{1}3$) and the ternary (Mn${}_{3}$IrSi, cP20, 198, $P\;2_{1}3$). In both cases, the 8c Wyckoff position (u,u,u) of the A13 structure is split into two 4a positions of space group 198 with different site occupations. Further, position 12d (1/8,u,1/4+u) becomes 12b. However, the splitting of the 8c position causes the change of symmetry from space group $P\;4_{1}32$ to $P\;2_{1}3$.

Another large number of compounds with chiral structure exists in the cubic space group 198, $P\;2_{1}3$. This group is not polar and the compounds do not exhibit pyroelectricity; however, they are optically active and can be piezoelectric. Famous in space group 198 are the B20 compounds, which will be dealt with in more detail. In addition, solid ammonia is in this space group as well. The B20 compounds exhibit not only a chiral crystalline structure but also a chiral magnetic order (for example MnSi and FeGe are hosting the skyrmions). Further compounds with the B20 prototype structure (FeSi, cP8, 198, $P\;2_{1}3$) are AuBe, CoGe, CoSi, CrGe, CrSi, FeGe, FeSi, HfSn, HfSb, MnGe, MnSi, NiSi, OsSi, PdAl, PdGa, PtAl, PtGa, PtMg, ReSi, RhGe, RhSi, RhSn, RuGe, RuSi, TcSi, and ZrSb.

These compounds with a B20 structure possess various different physical properties, which are for example:

- Semiconductivity: FeSi, OsSi, RuZ (Z=Si, Ge) [50]
- Kondo insulators [50]
- Magnetic order: MnZ, CoZ, (Z=Si, Ge), and FeGe as well as some mixed alloys (Fe${}_{1-x}$Co${}_{x}$, etc.)
- Superconductivity: AuBe, ReSi
- Topological types: CoSi, RhSi, PtAl

**Figure 6:**
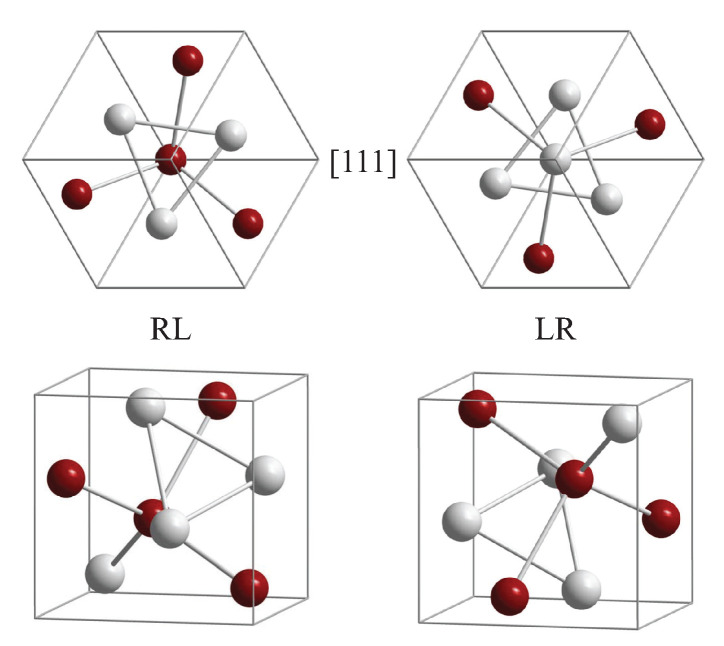
The B20 structure of FeSi. The enantiomorphous pair RL and LR is shown with $\left(u_{\mathrm{Fe}},u_{\mathrm{Si}}\right)=\left(0.3858,0.094\right)$ and (0.6142,0.906), respectively. RL implies that Fe atoms are positioned in an R type and Si in an L type structure and the opposite for LR. Both structures are shown for views along the [111] axes and arbitrary axes. Fe (or Si) atoms in the triangles of the [111] view are in the same (111) plane; these planes appear at a distance of $\left(2-u_{i}\right)/\sqrt{3}$ from the origin. Atoms in the center, on the [111] axis, are in a different plane $u_{i}\sqrt{3}$ away from the origin. See Figure 3 for the positions of the screw axes. Connections between atoms are drawn for better visibility and may not be confused with bonds.

**Figure 7:**
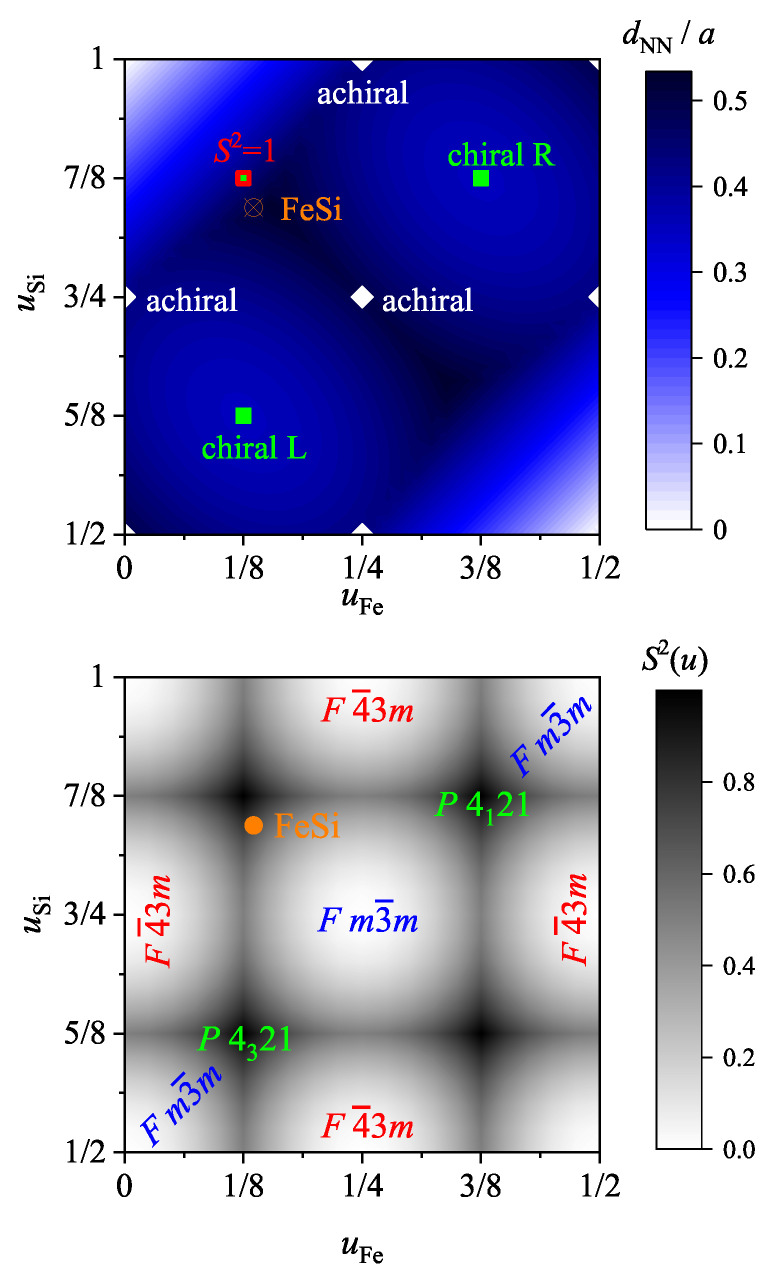
Chirality and nearest neighbor distances of the compounds with FeSi structure. Shown are on top the nearest neighbor distances and on the bottom the continuous chirality measure $S^{2}\left(u_{\mathrm{TM}},u_{\mathrm{MG}}\right)$ for space group 198 with two 4a Wyckoff positions occupied (B20 compounds). The values for FeSi (LR), the space groups of the achiral structures where S=0, or the chiral space groups where S=1 are assigned in the graph. Important note: The borderline parameters $\left(u_{\mathrm{TM}},u_{\mathrm{MG}}\right)=\left(0,1\right)$, and (1/2,1/2) are not possible as is evident from the graph of the nearest neighbor distances. Note that the largest possible nearest neighbor distance in B20 compounds is $d_{\mathrm{NN},\max}/a=\sqrt{3}/\left(1+\sqrt{5}\right)$.

The structure of the prototype (FeSi, cP8, 198) is shown in Figure 6. A FeSi racemate contains the same amounts of an enantiomorphous pair of structures with opposite chirality sense. Here, the enantiomorphous pair is given for example by the pairs RL and LR with $\left(u_{\mathrm{Fe}},u_{\mathrm{Si}}\right)=\left(0.3858,0.094\right)$ and (0.6142,0.906), respectively. The *u* parameters are provided for the standardized crystallographic data. RL implies that the sublattice of the Fe is of R type and the Si sublattice is of L type. In contrast, the opposite is true for LR. (Some work uses alternatively *R* and *S* (from Latin sinistram = left) whereas for molecules often *D* (from Latin dexter) and *L* (from Latin laevus) are used (see also [26]). In other cases, the two enantiomers of the B20 structure are assigned by A and B [51,52].) Both structures are shown for views along the [111] axes and along arbitrary axes. The Fe (or Si) atoms observed in the triangles of the [111] view are in the same (111) plane, whereas atoms in the center, on the [111] axis, are in different planes. In addition, the projection of the positions onto the [111] axis is $p_{111}=1-u$ for the off-axis atoms and $p_{0}=u\sqrt{3}$ for the on-axis atoms at (u,u,u). Owing to the different chiral character of the two sublattices, the structure of most of the B20 compounds cannot be directly assigned a particular handedness even for the case of enantiopure single crystals. Thus, the handedness would only be definite if one of the species occupied high symmetry sites of the cubic lattice whereas the other one is located on sites with lower symmetry resulting in a screw axis. An example would be $\left(u_{\mathrm{Fe}},u_{\mathrm{Si}}\right)=\left(0<u_{\mathrm{Fe}}<1/4,1/2\right)$ with the highest chirality measure of 50% at (1/8,1/2) at an Hausdorff distance of 1/8 (compare also Figure 4 and Figure 7). In such cases, one still stays in space group number 198.

The continuous chirality measure is defined by Equation (2). Here, the positions $p_{i}$ are defined by the position parameter *u* of the transition metal ($u_{\mathrm{TM}}$) and main group element ($u_{\mathrm{MG}}$) such that n=2. $p_{0}$ denote the values for the closest achiral structure either with space groups $F\;m\bar{3}m$ (225) or $F\;\bar{4}3m$ (216) that appear for position parameters $u_{0}=0$, 1/4, 1/2, 3/4, or 1. The closest implies that $p_{0}$ is chosen such that the norm ${∥p_{i}-p_{0}∥}^{2}$ is at its minimum. Further, for parameter pairs $\left(u_{\mathrm{TM}},u_{\mathrm{MG}}\right)=\left(0,1/2\right)$, (1/4,3/4), or similar, the rocksalt (NaCl type) structure with space group 225 is observed, whereas the pairs $\left(u_{\mathrm{TM}},u_{\mathrm{MG}}\right)=\left(0,1/4\right)$, (1/2,3/4), or similar result in the zincblende (ZnS type) structure with space group 216.

Moreover, $S^{2}\left(G\right)$ is normalized by the structure that appears when both *u* parameters attain the paired values (1/8, 5/8), or (3/8, 7/8) resulting in $\min({∥p_{e}-p_{0}∥}^{2})=3/64$. In contrast to the simple cubic problem with a single site of Section 4 above, the enantiomorphous pair of space groups (212), (213) appears for these *u* parameters only when the difference between the two parameters is 1/2 and thus both sublattices exhibit the same handedness. This implies that the compounds adopt at $S^{2}\left(G\right)=1$ not necessarily one of the chiral space groups, as explained above.

**Figure 8:**
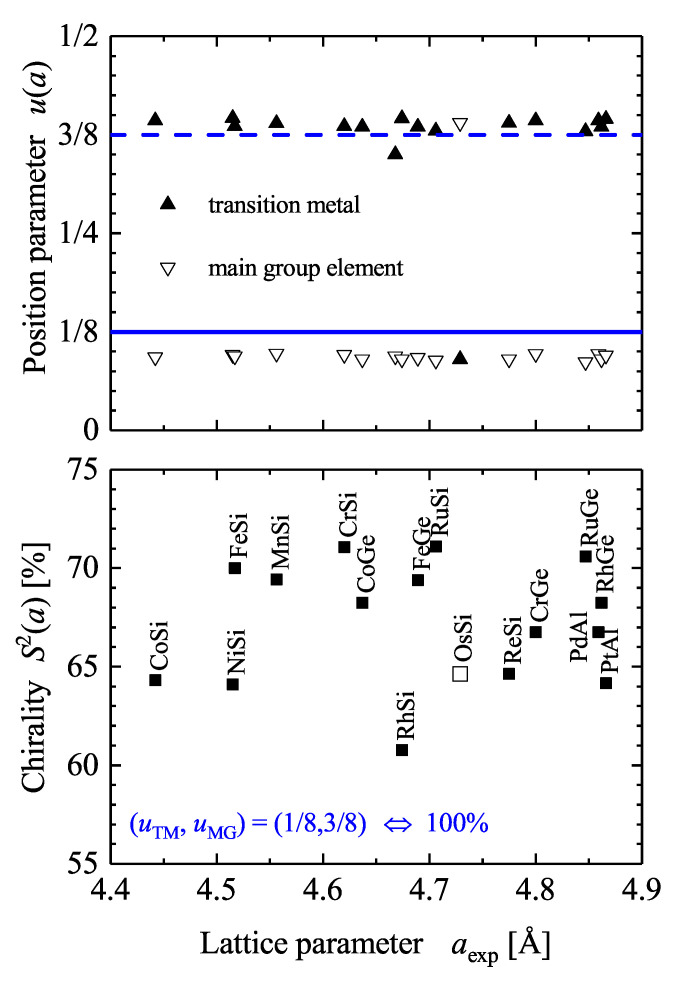
Chirality of the B20 compounds. Continuous chirality measures of B20 compounds where the complete structure determination is reported in Pearson’s database [20]. The full and dashed lines mark *u* values of 1/8 and 7/8, respectively.

Figure 8 summarizes the position parameters *u* and the resulting continuous chirality measure $S^{2}$ for various B20 compounds reported in literature. It is evident that the chirality measure of the considered compounds is in the range of 60 to 75% but does not depend on the lattice parameter *a*. Thus, this reflects the fact that the *u* parameters are not that dependent on the lattice parameter. The average position parameters for the compounds shown in Figure 8 are ${\bar{u}}_{\mathrm{TM}}=0.39$ and ${\bar{u}}_{\mathrm{MG}}=0.093$. In addition, the average chirality measure is 66.5%.

α-N${}_{2}$ (prototype: [N${}_{2}$], cP8, 198, $P\;2_{1}3$) has a similar chiral structure. Owing to the appearance of N${}_{2}$ molecules it is slightly different from the B20 compounds. The *u* parameters of α-N${}_{2}$ are (0.180, 0.2878) [53]. α-CO has the same structure with position parameters ($u_{C},u_{O}$) = (0.292, 0.183) [54]. Both systems may be viewed as chiral molecular solids.

For the body-centered cubic space group 199 in the Pearson database [20], the binaries CoLa and CoU (CoU, cI16, 199, $I2_{1}3$) can be found. However, the 1:1 composition of CoLa is not stable, but CoLa${}_{x}$ is stable [55,56]. In addition, various oxides with the samarium oxide structure (Sm${}_{2}$O${}_{3}$, cI80, 199, $I2_{1}3$) adopt this space group as well.

There are also many simple ternary compounds found in space group 198. They adopt the NiSSb (Ullmanite) or $F0_{1}$ structure. Two equivalent prototypes are assigned to that structure, namely (NiSbS, cP12, 198) and (ZrSO, cP12, 198). In principle, the structure is based on the $C1_{b}$ or (CuMgSb, cF12, 216) structure of the XYZ Heusler compounds with 1:1:1 stoichiometry. However, here the atomic positions are shifted away from the high symmetry positions of the $C1_{b}$ structure.

The $F0_{1}$ structure hosts the oxides ZrSO and HfSO with 16 valence electrons in the cell. Furthermore, based on elements from the Co group 9 (VIII), we obtain with 16 valence electrons: IrBaP and IrSrP, or with 20 valence electrons: CoAsS, RhBiSe, RhSbS, RhSbSe, RhPSe, RhSbS, RhSbSe, IrBiS, IrBiSe, IrSbSe, IrPSe, IrSbS, IrSbSe, and IrSbTe.

Based on elements from the Ni group 10 (VIII) we obtain with 16 valence electrons: PdBaSi, PdSrSi, PtCaSi, PtBaSi, PtSrSi, and PtBaGe, PtSrGe; with 17 valence electrons: PtBaAs and PtBaP; with 21 valence electrons: NiAsS, NiBiSe, NiSbS, NiSbSe, NiPS, PdAsS, PdAsSe, PdBiSe, PdSbS, PdSbSe, PdSbTe, PtBiSe, PtSbS, and PtSbSe.

However, the Pearson data base [20] reports certain compounds that do not exist in the $F0_{1}$ structure, such as PtCrSb and PtFeSb [57]. To date, no chiral compound that has a typical Heusler composition with two different 3d transition metals and one main group element has been reported.

The only known compounds with one main group and two transition metal elements are LaRhSi and LaIrSi. Further, rare earth-containing compounds reported with $F0_{1}$ structure are CeIrSi, NdIrSi, and PrIrSi with $16+n_{4f}$ valence electrons, where $n_{4f}$ is the number of 4f electrons, and further EuIrP, EuPdSi, EuPtSi, and EuPtGe with $17+n_{4f}$ valence electrons. Among those, LaRhSi and LaIrSi are known to be superconductors with transition temperatures of 4.35 and 2.3 K, respectively [58]. In the same work [58] it was proposed that the magnetic structure of NdIrSi is probably non-collinear.

**Figure 9:**
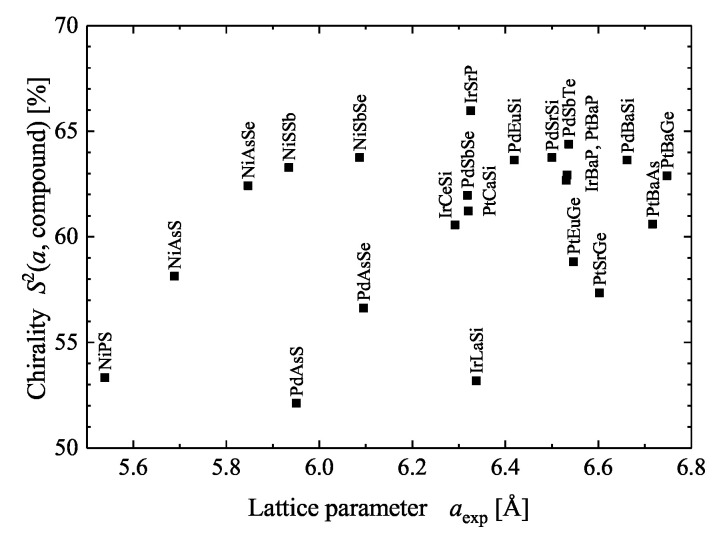
Chirality of the $F0_{1}$ compounds. Continuous chirality measures of $F0_{1}$ compounds where the complete structure determination is reported in the Pearson database [20].

Figure 9 summarizes the continuous chirality measure of various $F0_{1}$ compounds. It is evident that the chirality of the considered compounds is in the range of 52 to 66% but does not depend on the lattice parameter, as was already discussed for the B20 compounds. Further, an ideal $F0_{1}$ structure will still belong to space group 198, for example with the three *u* parameters 1/8, 3/8, and 5/8, whereas the achiral case with space group $F\;\bar{4}3m$ appears exemplary for $u_{1,2,3}$ parameters of 0, 1/4, and 1/2. Moreover, in many cases the *u* parameter of the transition metal atom is close to zero; that is, its contribution to the chirality measure that is defined by the positions of the main group elements is minimal. The set of *u* parameters with 0, 3/8, and 5/8 still results in a chiral structure, albeit with a reduced continuous chirality measure of 2/3. However, the small contribution of the transition metals to the chirality measure is different from the B20 compounds, where they are mainly responsible for the size of the chirality measure and Hausdorff distance.

The high-pressure structure of PdF${}_{2}$ is also reported to appear in this space group (PdF${}_{2}$, cP12, 198, $P\;2_{1}3$). Under moderate pressure (p>1.4 GPa) the structure of PdF${}_{2}$ changes from rutile (TiO${}_{2}$, tP6, 136, $P\;4_{2}/mnm$) to a C2 pyrite (FeS${}_{2}$, cP12, 205, $P\;a\bar{3}$) or to the $F0_{1}$ ullmannite type structure, that is, from an achiral form to a chiral one. The fluorine site at u=0.3431 of the C2 structure splits into two positions with $u_{1}=0.344$ and $u_{2}=0.658$ at a nearly unchanged lattice parameter of $a_{\exp}=5.329$ Å. In other words, the two fluorine atoms are in $F0_{1}$ on screw axes with opposite chirality sense (see Figure 4). Pd is placed at the achiral site with $u_{0}=0$ in both structures, C2 and $F0_{1}$. Therefore, the chirality in the $F0_{1}$ structure is defined solely by the fluorine atoms. Further, the splitting of the fluorine positions away from the one observed in the C2 structure is very small. Here the C2 structure is the closest achiral structure and not C1 (CaF${}_{2}$, cF12, 225, $F\;m\bar{3}m$) or $C1_{b}$, which results in very small chirality measures. Compared to the two achiral structures, the Hausdorff distances are h(C2)=0.001 and h(C1)=0.093. This demonstrates the necessity of determining and always using the closest achiral structure when calculating chirality measures.

### 5.3. Compounds with Tetragonal Chiral Structures

WOBr${}_{4}$ and WOCl${}_{4}$ are interesting tetragonal systems as they have a rather simple structure (WOCl${}_{4}$, tI12, 79) with only three different atomic positions. This structure is chiral as well as polar and belongs to space group I4. Tungsten occupies a high symmetry 2a position at (0,0,0) of the tetragonal lattice, whereas the oxygen (0,0,u) and halogen (x,y,z) atoms occupy Wyckoff positions 2a and 8c with free parameters. The experimentally reported free parameters of WOCl${}_{4}$ for oxygen and chlorine are u=0.545 and (x,y,z)=(0.0669,0.2584,0.0789), respectively. In addition, the symmetry remains unchanged if the oxygen positions are changed while the chlorine positions are maintained. A close centrosymmetric structure can be easily determined by setting u=1/2, x=z=0, and y=1/4. The resulting structure has I4/mmm (139) symmetry and is achiral and non-polar. Another achiral and non-polar structure is found by setting u=1/2 and only z=0 resulting in I4/m (87). Furthermore, the chirality can be removed while maintaining the structure as non-centrosymmetric, when setting the chlorine parameters to (x,y,z)=(0,1/4,0), but keeping the *u* parameter of the oxygen atoms. Consequently, the resulting structure possesses I4mm (107) symmetry and is no longer chiral although still polar. The chirality measures for WOBr${}_{4}$ and WOCl${}_{4}$ are presented in Table 7. The Hausdorff distance is dominated by the oxygen position, whereas *s* depends on both, O and halogen positions. The closest achiral structure is the one with the smallest continuous chirality measure and thus I4 in both cases. This example shows the manner in which to select the achiral structure for comparison.

### 5.4. Compounds with Hexagonal or Trigonal, Chiral Structures

Several sets of chiral space groups appear for hexagonal structures. Among the interesting chiral materials is quartz SiO${}_{2}$ as its C40 structure belongs to the chiral space group (180). This space group, $P\;6_{2}22$, has an enantiomorphous partner, namely $P\;6_{4}22$. Further, the prototype of the C40 structure is CrSi${}_{2}$ (hP9, $P\;6_{2,4}22$, 180, 181) and several transition metal (*T*) silicides *T*Si${}_{2}$ crystallize with the same structure. Additional compounds with this structure are CrSi${}_{2}$, VSi${}_{2}$, NbSi${}_{2}$, TaSi${}_{2}$, MoSi${}_{2}$, WSi${}_{2}$, VGe${}_{2}$, NbGe${}_{2}$, TaGe${}_{2}$, WAl${}_{2}$, HfSn${}_{2}$, and NiMg${}_{2}$. SiO${}_{2}$ is a wide band gap (>5.9 eV) insulator and CrSi${}_{2}$ is a narrow band gap (0.35 eV) semiconductor with trivial topology [59]. The remaining compounds with a C40 structure are metals or semimetals.

**Figure 10:**
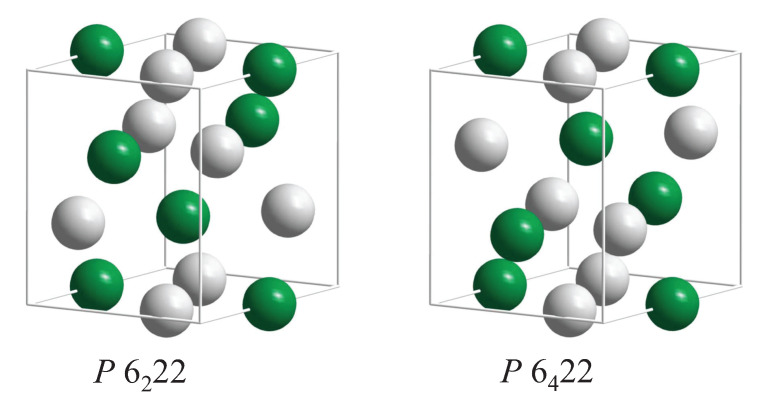
The CrSi${}_{2}$ structure of quartz (SiO${}_{2}$) and transition metal C40 compounds. The enantiomorphous pair of structures in the chiral space groups $P\;6_{2}22$ (180) and $P\;6_{4}22$ (181) are shown. Transition metal atoms (Cr) are green (dark), and the main group elements (Si) are shown in grey (light). (For quartz, green atoms correspond to Si and grey ones to O).

The atoms of compounds with C40 structure are placed on Wyckoff positions 3c (1/2, 0, 0) and 6i (u,2u,0) (or 3d (1/2, 0, 1/2) and 6j (u,2u,1/2) (see Figure 10). In the prototype CrSi${}_{2}$, u=0.16577 can be observed. However, the structure becomes achiral when the Cr atoms are placed at 3a,b (0, 0, *w*) and simultaneously the Si atoms on 6i,j (1/3, 2/3, *w*), where w=0 or 1/2. The resulting achiral structure belongs to space group P6/mmm (191). Table 8 summarizes the chirality of the compounds with C40 structure where the complete structure was determined according to the Pearson database [20].

The Hausdorff distance for the atoms on the 3c position is fixed because this position has no free parameter. The former has the value $h_{1}=1/2$. However, the value of the atoms in the 6i position varies and is $h_{2}=u\;\sin\left(60^{\circ }\right)=u\sqrt{3/4}$ for 0≤u≤1/2 (u=0 or 1/2 cannot be reached, because the distance between atoms cannot vanish). Consequently, the largest value of the continuous chirality measure attained is $S^{2}\left(u\right)=1$. The normalized chirality measure of CrSi${}_{2}$ is $S^{2}=27$%.

Cinnabar (HgS, hP6, $P\;3_{1}21$, 152) with a B9 structure is the prototype for a set of chiral binary compounds within the chiral space group 152. HgS, HgSe, HgTe, HgO, and partially ZnTe adopt this structure. Moreover, the topological status of HgS and the other mercury compounds in space group 152 is trivial [59]. Sometimes the prototype is assigned to CdTe, however, CdTe also exists in other structures. In addition, the structure was also observed in a high-pressure phase of GaAs. Furthermore, at high pressure (11.2 GPa) a chiral phase of ZnTe (ZnTe, hP6, $P\;3_{1}$, 144) was reported that is derived from the Zincblende structure.

Both positions (3a ($u_{1},\;0,\;1/3$) and 3b ($u_{2},\;0,\;5/6$)) of the hexagonal HgS structure have free parameters $u_{i}$ that determine the chirality. The continuous chirality measure of the cinnabar structure is $S^{2}=2\left({u^{\prime }}_{1}^{2}+{u^{\prime }}_{2}^{2}\right)$ where the position parameters are reduced to $u^{\prime }=\min(u,|1-u|)$. Consequently, it becomes $S_{\mathrm{HgS}}^{2}=0.637$ using $u_{\mathrm{Hg}}=0.28$ and $u_{S}=0.51$. Further, $u_{1}=u_{2}=1/2$ results in the chiral space group $P\;6_{4}22$ (181), whereas $u_{1}=u_{2}=0$ results in P6/mmm (191) and thus the structure becomes achiral.

Before continuing with 2-dimensional systems we would like to point to an overview of chiral metals given by Riva [60], reporting mostly intermetallic compounds with many more atoms in the cell than considered in this study. The chiral oxides were compiled very complete by Halasyamani and Poeppelmeier [61]. Yiwen Li et al. [62] focused on chiral transition metal oxides. Recently, the properties of chiral, nanostructured materials and their applications [63] have been reviewed [63].

## 6. Chirality at Solid Surfaces

The occurrence of surfaces has drastic effects on symmetry. The translational symmetry along the surface normal is broken and inversion as well as horizontal mirror operations in the surface are removed as the latter two would exchange bulk and vacuum. Moreover, the symmetry is no longer described by the 3-dimensional space groups and thus the wallpaper groups (see Section 3) are used instead. With respect to chirality, four different situations may arise:

1. achiral bulk with a chiral surface,
2. chiral bulk with an achiral surface,
3. chiral bulk with a chiral surface,
4. achiral bulk with an achiral surface.

The last case of an achiral bulk with an achiral surface is the most trivial and will not be considered here. In addition, surfaces may also be modified to become chiral via reconstruction or adsorption of atoms, molecules, or chiral molecules. These cases will also not be included in the discussion. We consider certain simple examples. The importance of chiral inorganic crystalline surfaces for chiral selection and discrimination was reviewed by Hazen and Sholl [64]. The chiral geometry of the surfaces of metal nanocrystals was reviewed by the group of Ki Tae Nam [65]. The case of two-dimensional chiral molecular assembly on solid surfaces was reviewed by Chen et al. [66].

### 6.1. Achiral Bulk with Chiral Surface

The most prominent examples for the first case are the high index surfaces of elemental metals. Particular examples are the fcc(hkl) surfaces with (hkl)=(531), (643), and (874). Typical for this type of surface is the occurrence of narrow (111) oriented terraces with high densities of steps and kinks. The kinks serve as chiral centers. The enantiomorphous pair of surfaces (hkl) and $\left(\bar{h}\bar{k}\bar{l}\right)$ with h≠k≠l≠0 are denoted by ${\left(hkl\right)}^{R}$ and ${\left(hkl\right)}^{S}$, respectively. The upper indices assign the clockwise (*R*) or anticlockwise (*S*) decrease of the atom density about the kink atoms [67]. $fcc(h\bar{k}\bar{l})$, $fcc(\bar{h}k\bar{l})$, and $fcc(\bar{h}\bar{k}l)$ are equivalent to fcc(hkl).

**Figure 11:**
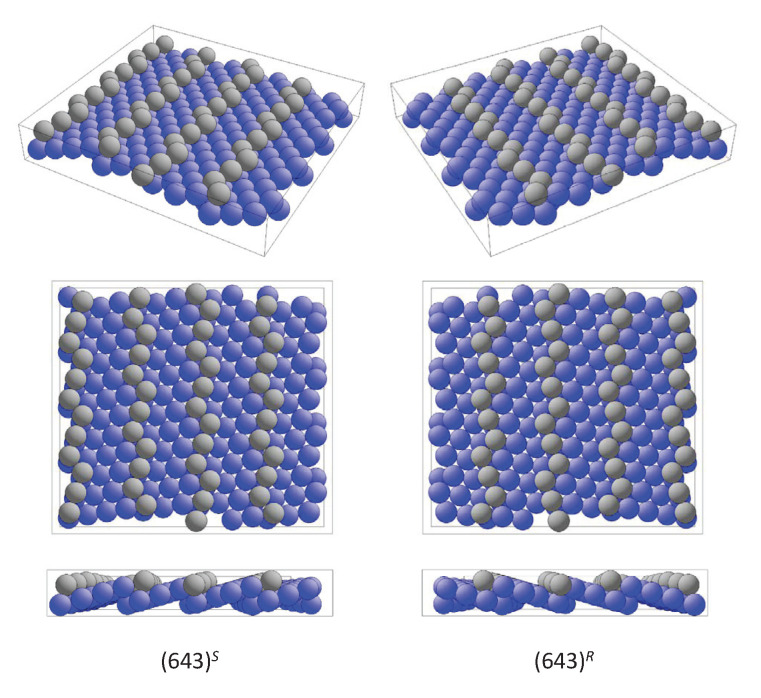
The chiral fcc(643) surface. The two modifications $fcc{\left(643\right)}^{S}$ and $fcc{\left(643\right)}^{R}$ with opposite handedness from different perspectives are shown. Different colors are used to better distinguish edge and kink atoms from those on terraces.

Figure 11 shows the enantiomorphous pair of $fcc{\left(643\right)}^{S,R}$ surfaces. Such high index surfaces are observed for Au, Ag, Cu, or Pt and, in particular, many of the high index Pt surfaces are very stable. The edge and kink sites are drawn in different colors to allow a better comparison of the handedness. However, the surface atoms are not coplanar as is evident from the side view. Moreover, the plane group symmetry of the surface is p2, thereby confirming its chirality.

Chiral surfaces appear in fcc as well achiral sc, bcc, or hcp structures, see [68,69]. Similar to the case of fcc structures, chiral bcc(hkl) surfaces are characterized by three inequivalent Miller indices (h≠k≠l≠0) and those fcc(hkl) and bcc(hkl) surfaces with identical Miller indices exhibit the same handedness. In certain cases, the indices *S* and *R* are replaced by *D* and *L*, respectively, where the latter are often used to characterize chiral molecules as well. However, the case of hcp surfaces is more complex, and a complete description of chiral and achiral surfaces of the elemental metals with bcc, fcc, or hcp structures was reported by Jenkins and Pratt in Ref. [68].

### 6.2. Surfaces of Chiral Bulk Materials

The question that arises is whether the surfaces of chiral bulk materials also need to be high indexed as in the case of achiral structures. Certain special projections for the space groups mentioned in previous sections are listed in Table 9. The remaining space groups may be found in Ref. [22]. It is obvious that certain projections along low index, high symmetry directions of chiral structures are achiral although the symmetry of the bulk is described by a chiral space group. However, there also exist certain low indexed projections that are chiral. For example, in space group 198 the symmetry of the projection along [111] belongs to the plane group p3 and thus, this projection is chiral.

The projections are ideally plane 2D structures without an extension in 3D space. In contrast, solid surfaces have an extension also in the direction perpendicular to the plane defining the surface. The difference lies in the fact that the periodicity along the surface normal is broken. Regarding binary or higher compounds, both the symmetry and the termination of the surface are vital and the element that defines the surface layer is important.

**Figure 12:**
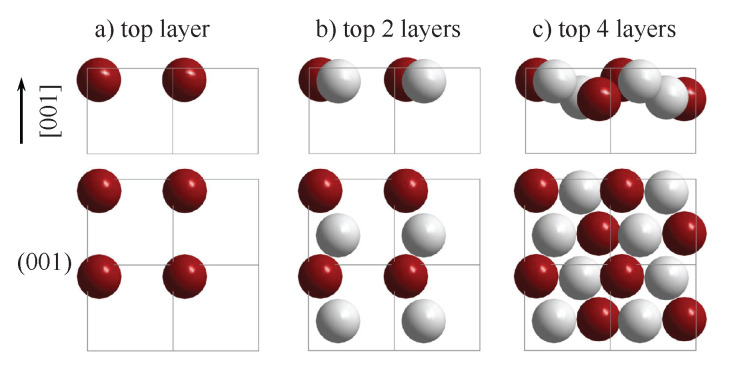
The FeSi(001) surface. The evolution of the surface structure and symmetry is shown by increasing the number of layers in (**a**–**c**). The upper row shows the side view and the lower row the top view. Fe atoms are drawn in red (**dark**) and Si atoms in grey (**light**).

The appearance of chiral surfaces of a chiral material will be illustrated for the example of space group 198 using the FeSi(001) surface. It is assumed that the surface is terminated by the Fe layer. This top layer exhibits a fourfold symmetry (p4mm) as is expected for a cubic crystal (Figure 12a). Further, the first Si layer is only slightly below the top Fe layer (Figure 12b), and removes the fourfold rotational symmetry as well as the mirror planes. Moreover, the surface appears to be closed when including four layers (Figure 12c). In the projection, the Fe and Si atoms appear to follow each a zig-zag line, and similar to the chiral, high index fcc surface, the atoms forming the closed surface are not coplanar, although kinks that serve as chiral centers do not appear. In reality, the structure comprises simple atom rows of different heights that are shifted with respect to each other.

**Figure 13:**
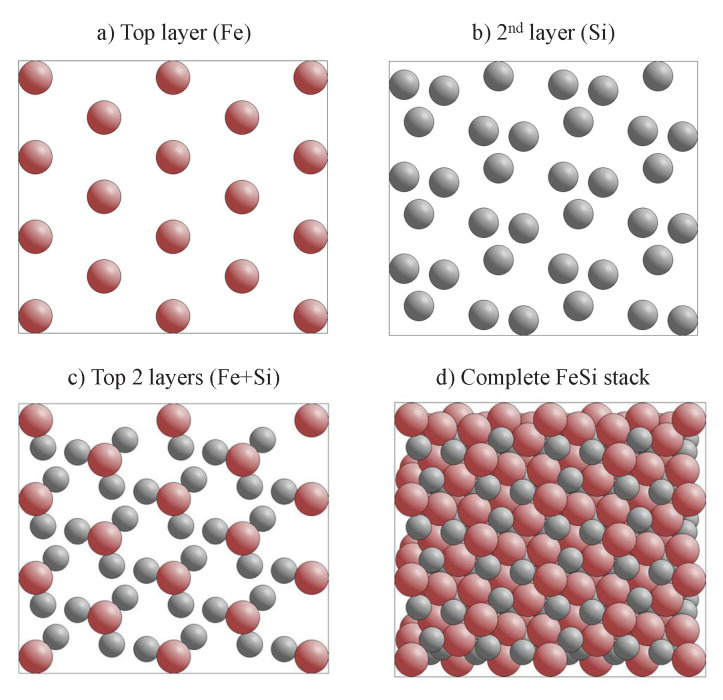
An Fe terminated FeSi(111) surface. The evolution of the surface structure and symmetry are shown for increasing number of layers in (**a**–**d**), assuming that the surface is terminated by Fe with layer type A (see text).

The evolution of the FeSi(111) surface structure is illustrated in Figure 13. The starting point is the Fe atom at $\left(u_{\mathrm{Fe}},u_{\mathrm{Fe}},u_{\mathrm{Fe}}\right)$ as center of the topmost layer in Figure 13a. This layer (type *A*) exhibits a sixfold rotational symmetry (p6mm) and has a hexagonal cell with a basis of one atom. It is followed by the Si layer (type *B*) shown in Figure 13b, whose cell has the same shape but with a basis of three atoms, resulting in a threefold symmetry. The orientation of the triangles of atoms depends on the position of the atoms in the three-dimensional cubic cell. The combination of these first two layers already results in the chiral p3 symmetry shown in Figure 13c. The completed chiral surface structure is shown in Figure 13d. Similar to the case of the FeSi(001) surface, no chiral centers were observed; however, the threefold rotational axes serve as chiral axes. Moreover, four different types of surfaces depending on the termination are expected: Fe type *A*, Si type *A*, Fe type *B*, or Si type *B*. The positions of the layers with respect to the origin along [111] are $u_{\mathrm{Fe}}\sqrt{3}$, $\left(2-u_{\mathrm{Si}}\right)/\sqrt{3}$, $\left(2-u_{\mathrm{Fe}}\right)/\sqrt{3}$, and $u_{\mathrm{Si}}\sqrt{3}$.

The surfaces of opposite sites of a chiral are not completely equivalent because of the lack of inversion and mirror symmetries. For example, the four-fold (001) and ($00\bar{1}$) in space group $P\;2_{1}3$ are not equivalent, in contrast to the (001) surfaces of a W type body-centered cubic structure with space group $I\;m\bar{3}m$. Consequently, this affects the measurements that are surface sensitive. For a full characterization of surfaces of chiral crystals, at least four measurements may be necessary, which are two each for the opposite surfaces of an enantiomorphous pair of crystals.

**Figure 14:**
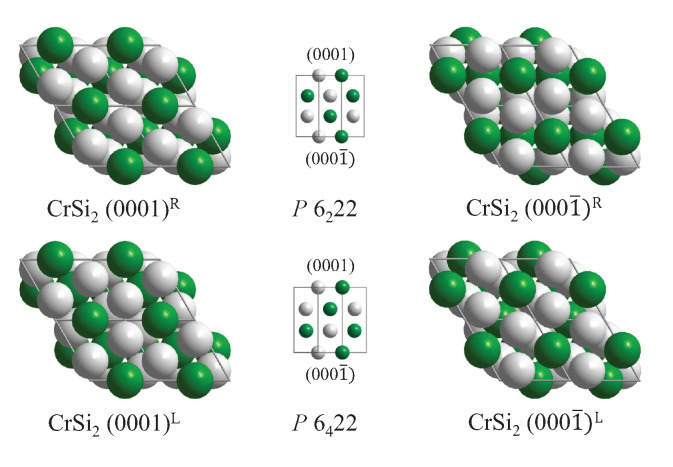
Different CrSi${}_{2}$(0001) surfaces and their ($000\bar{1}$) counterparts. The upper row shows the right-handed and the lower row the left-handed crystal, with the indicated space group. The stacking order is sketched in the middle using [$11\bar{2}0$] as viewing directions.

As a final example, the four different (0001) type surfaces of the chiral CrSi${}_{2}$ structure (compare Figure 10) are illustrated in Figure 14.

Upon comparing the chiral surfaces of chiral and achiral structures it is evident that the predominating difference is the appearance of chiral axes and centers in the first and second cases, respectively.

## 7. Electronic Structure and Chirality

This section discusses the relations between chirality and electronic structure. Simple prototypical elemental and binary systems, such as Se and FeSi were chosen. Details of the electronic structure calculations are summarized in Appendix A.

### 7.1. Electronic Structure of Se

**Figure 15:**
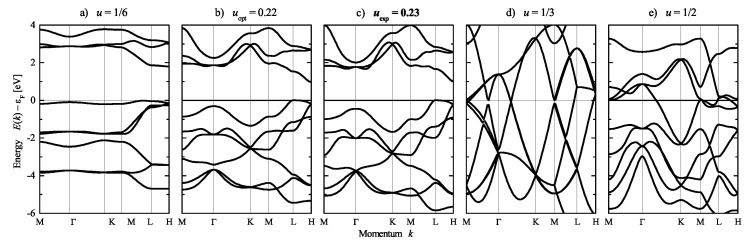
Electronic structure of chiral and achiral (u=1/3) Se. The band structures are calculated for variation of the chirality by changing the position parameter *u*. From left to right: (**a**) u=1/6 ($S^{2}=100$%), (**b**) u=0.22 (optimized) ($S^{2}=45.2$%), (**c**) u=0.23 (experiment) ($S^{2}=38.4$%), (**d**) u=1/3 ($S^{2}=0$), (**e**) u=1/2 ($S^{2}=100$%). The corresponding chirality measures are given in brackets (compare also Figure 5). Note that the parameters u=1/3 ($R\;\bar{3}m$) and u=1/2 ($P\;6_{4}22$) result in different space groups and symmetry. Calculations are for variation of the position parameter at optimized lattice parameters.

Se exhibits various different crystal structures [20]. Here, the chiral γ-Se structure is considered with respect to its electronic structure. The calculated band structure of Se is shown in Figure 15, where the calculations are based on the crystal structure with space group $P\;3_{1}21$. Subsequently, the position parameter *u* and thereby the chirality $S^{2}$ was varied. Further, the lattice parameters *a* and *c* were optimized for each $u\neq u_{exp}$ to avoid unphysically small nearest neighbor distances. Herein, $u_{opt}$ corresponds to a full structural optimization for *a*, *c*, and *u*. The variation of the chirality $S^{2}$ with *u* was already shown in Figure 5. Moreover, the calculations for the opposite handedness using the enantiomorphous space group $P\;3_{2}21$ result in identical band structures, which also includes space groups $P\;6_{2}22$ and $P\;6_{4}22$.

Se was determined to be a semiconductor at the experimental lattice parameters including the position parameter (u=0.23 with $S^{2}=0.38$) as well as at optimized lattice and position parameters. For u=1/6, $S^{2}=1$ was obtained, and the band gap was the largest. However, Se becomes metallic in the achiral structure with u=1/3 and $S^{2}=0$ and is also metallic at u=1/2 where the chiral space group $P\;6_{4}22$ is reached.

Considering the band structure at the experimental lattice parameters (Figure 15c), a highly degenerated state appears at Γ approximately 4 eV below the Fermi energy. A detailed analysis shows that there is a small splitting of only 11 meV between the upper a1 and the lower *e* states. However, the small variation of the *u* parameter from 0.23 to 0.22 already results in a much larger splitting of 266 meV in the optimized structure (Figure 15b). Simultaneously, another highly degenerate state at *K*, approximately 3 eV below $\epsilon_{F}$, stays rather unoffended.

However, a dependence of the electronic structure on the (free) *u* parameter is also observed in achiral systems. Therefore, it is concluded that the general character of the band structure E(k,u) depends predominantly on the position parameters (*u*), with the dependence on the chirality $S^{2}\left(u\right)$ or handedness being only indirect.

### 7.2. Electronic Structure of FeSi and Other Compounds with B20 Structure

**Figure 16:**
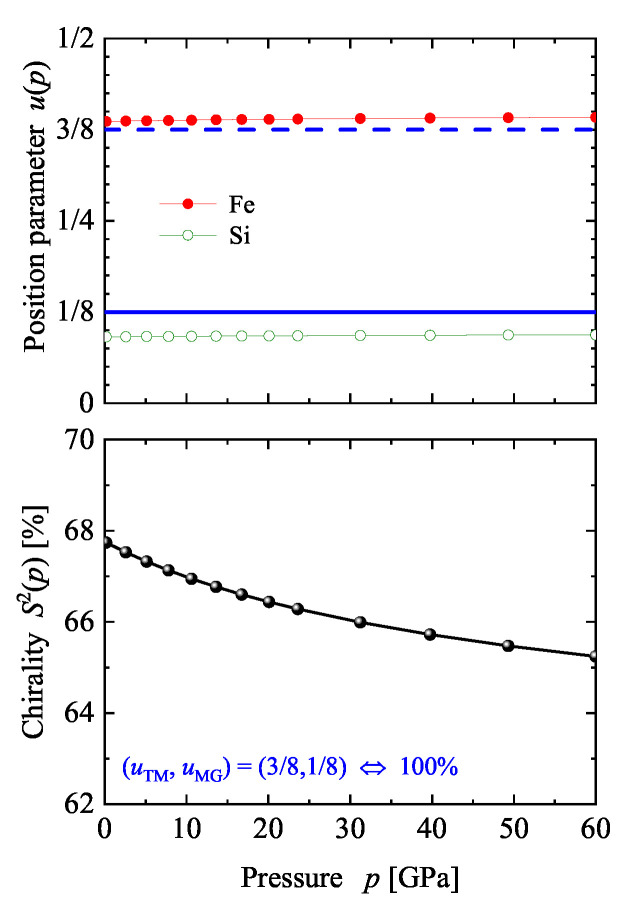
Pressure dependence of the chirality of FeSi. The position parameters (symbols connected by lines) for Fe and Si and the continuous chirality measure $S^{2}\left(p\right)$ are shown. The position parameters resulting in $S^{2}=1$ are marked in the upper part by full (1/8) and dashed (3/8) lines (blue).

Before discussing certain details of the electronic structure of FeSi, the problem of “how to change the chirality’ of a given structure is addressed. If the atomic positions are fixed by the composition then an external parameter that is able to change these positions in a controlled manner is required. One external parameter is pressure. Here, hydrostatic pressure was simulated in the calculation by changing the volume away from its equilibrium to smaller values. Thereafter, the pressure was determined using the equation of state. The results obtained for the prototype FeSi are illustrated in Figure 16. The position parameters remain, with a change of approximately 1…3%, nearly unaffected while the chirality decreases from 68 to 65% with increasing pressure (0≤p≤60 GPa). In addition, the band gap decreases by only 10 meV when applying a pressure of approximately 60 GPa. Consequently, much higher pressures (up to 500 GPa) would be needed to approach the ideal position parameter of Vocadlo et al. [70].

**Figure 17:**
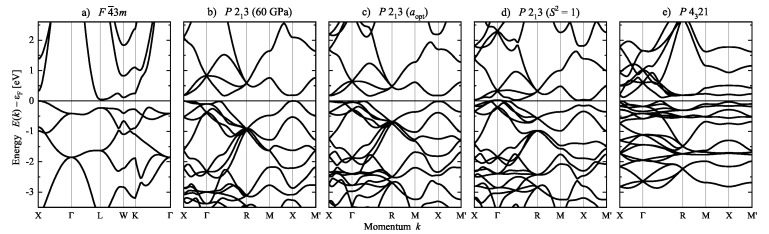
Band structure of FeSi, calculated for variation of the chirality by changing the position parameters $u_{\mathrm{Fe}}$ and $u_{\mathrm{Si}}$. From left to right: (**a**) $F\;\bar{4}3m$ achiral, (**b**) $P\;2_{1}3$ at 60 GPa pressure, (**c**) $P\;2_{1}3$ optimized, (**d**) $P\;2_{1}3$ with $S^{2}=1$, and (**e**) $P\;4_{3}21$ also with $S^{2}=1$. Calculations are for variation of the position parameter at optimized lattice parameter *a* for each structure. Note that the Δ direction ($\bar{\Gamma X}$) has in space group 198 only a 2-fold rotational symmetry, therefore, the perpendicular directions $\bar{MX}$ and $\bar{XM^{\prime }}$ are not equivalent. (Please note the different Brillouin zone of the face-centered space group in (**a**)).

The calculated band structure of FeSi is shown in Figure 17 as a function of position parameters *u* and chirality measure. Similar to the case of Se, the band structures are identical when the chirality sense of the structure is reversed by applying mirror operations or inversion to the crystal structure. In addition, the lattice parameter *a* was optimized at fixed position parameters *u* to allow for a better comparison. The change in the *u* parameters result in rather large differences in the lattice parameters caused by the variation of the nearest neighbor distances. At the optimized lattice and position parameters, FeSi is a semiconductor with an indirect band gap of ≈170 meV. Further, in the achiral ZnS structure, the size of the band gap is reduced to ≈50 meV. Consequently, the compound becomes semimetallic with a small overlap of approximately 30 meV between valence and conduction bands when the *u* parameters are changed such that S=1. However, the space group $P\;2_{1}3$ is maintained. In the case of the structure with chiral space group $P\;4_{3}21$, the band gap is zero without an overlap of bands. Although not shown here, two other cases are also remarkable. FeSi would become metallic in the NaCl structure with space group $F\;m\bar{3}m$, while, an indirect band gap of approximately 150 meV is still retained when only Fe determines the chirality and $\left(u_{\mathrm{Fe}},u_{\mathrm{Si}}\right)=\left(1/8,1/2\right)$. Furthermore, the effect of pressure up to 60 GPa on the band structure is minimal.

RuSi, RuGe, and OsSi are also semiconductors, with their band structure being very similar to FeSi, provided the spin–orbit interaction is neglected. They were investigated theoretically in Ref. [71], where it was found that the RL type structure can be distinguished from LR via the electric polarization response to a magnetic field. However, this has yet to be verified experimentally. Recently, it was shown that the crystal symmetry can yield free fermionic excitations thereby giving rise to Fermi arcs in non-Weyl systems [72]. Consequently, a notable number of publications demonstrated that these excitations are found in the B20 compounds CoSi, RhSi, and PtAl [48,49,73,74]. The observed Fermi arcs are interesting because of their unusual Chern numbers (±2). The conduction band of FeSi reveals a four-fold degeneracy at the *R* point (at ≈0.5 eV above $\epsilon_{F}$). However, this point becomes six-fold degenerated when spin–orbit interaction is included. The appearance of the four- and six-fold degenerate states has been explained using theoretical methods in Ref. [72]. For CrSi, with fewer electrons occupied, a highly degenerate point at *R* coincides with the Fermi energy (at 0 eV), with the energies at the Γ point being above and below 0 eV. In addition, in Ref. [49] the B20 compound PtAl was discovered to be a topological metal that hosts four- and six-fold fermions with Chern numbers of ±2 resulting in long Fermi arcs.

Thus, it was found that the characteristic quantities of the electronic structure, namely the band structure and consequently the density of states, depend on the position parameters of the atoms in the crystal structure but are not indicative of chirality and handedness.

### 7.3. Berry Curvature and Chirality

In the previous section, the band structures E(k) were determined to be independent of the chirality sense of a crystal. They are the same in the enantiomorphous pair, although the symmetry elements of the Hamiltonian are different. This is because the important information on the wave functions arising from the chiral potential is not included in these quantities. Another quantity being related more directly to the wave functions is the Berry curvature [75,76] and it is expected to carry certain information on the chirality. Certain crystals lacking an inversion center are known to exhibit piezoelectricity (see Table 1), which is connected to the Berry phase. The sign of the Berry phase is changed when the structure is inverted. However, not all chiral materials are piezoelectric, and vice versa. Therefore, piezoelectricity cannot be used in all cases to distinguish between enantiomers. In particular, space group $P\;2_{1}3$ (198) provides piezoelectricity and thus, the compounds with B20 or $F0_{1}$ structure may be piezoelectric. However, piezoelectricity can only be measured for insulating materials, which requires usually large band gaps. Here, the influence of chirality and structural inversion on the Berry curvature instead of the Berry phase will be briefly investigated for FeSi.

The Berry curvature $\vec{\Omega }\left(k\right)$ as a function of the electron momentum *k* is defined in the pseudovector form by the equation [77]:

(3) $$\vec{\Omega }\left(k\right)=-ℑ\left(\langle {\vec{\nabla }}_{k}u_{k}\left|\times \right|{\vec{\nabla }}_{k}u_{k}\rangle \right)$$

where $|u_{k}\rangle =e^{-ik\cdot r}|\Psi_{k}\rangle$ are periodic Bloch wave functions, and *ℑ* assigns the imaginary part. Here, $\vec{\Omega }$ was calculated using Wannier functions based on first principle calculations of the electronic structure (see Appendix A).

**Figure 18:**
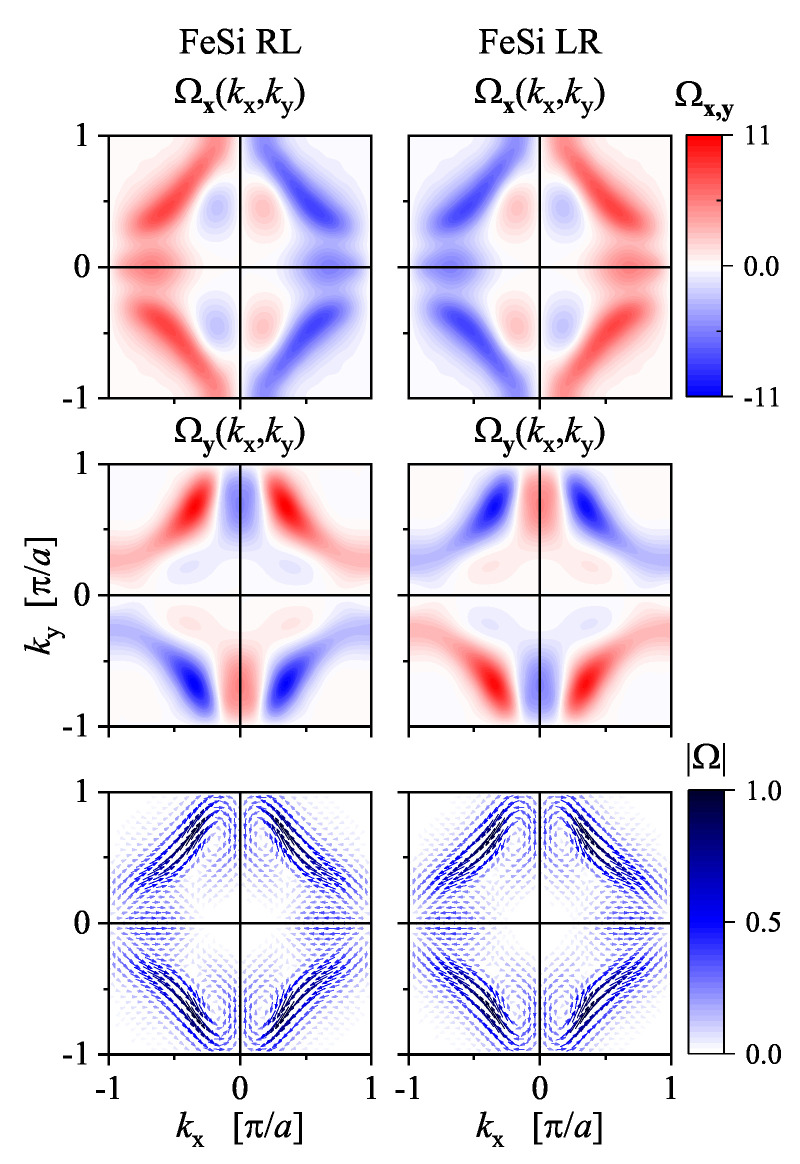
Berry curvature of FeSi, calculated for the two enantiomers of FeSi. The *x* and *y* components $\Omega_{x,y}\left(k_{x},k_{y}\right)$ in the (001) plane through the Γ-point ($k_{z}=0$) and the corresponding in-plane vector field $\vec{\Omega }\left(k_{x},k_{y}\right)$ are shown.

Figure 18 shows the calculated in-plane components of the Berry curvature of FeSi${}^{\mathrm{RL}}$ and FeSi${}^{\mathrm{LR}}$ in the (001) plane through the Γ-point ($k_{z}=0$). The *z*-component vanishes in this plane. The absolute value of the Berry curvature is the same and has an achiral $C_{2v}$ symmetry similar to the band structure. This is because of the sign and thus any phase information is lost when calculating it. However, the sign information is still present in the *x* and *y* components of Ω, to a certain extent. The *x* and *y* components have mirror planes that are absent in the crystal structure. Certain details and differences of the Berry curvature can be better observed from the plot of the vector field $\vec{\Omega }\left(k_{x},k_{y}\right)$, as shown in Figure 18. Remarkably, the rotational sense of the vector field distribution is opposite for FeSi${}^{\mathrm{RL}}$ compared to FeSi${}^{\mathrm{LR}}$, thereby demonstrating that the Berry curvature is a characteristic quantity of the electronic structure that is also sensitive to the handedness (or more general chirality sense) of a chiral crystal structure. The appearance of magnetic moments induced by a current as well as other Berry curvature-related gyrotropic effects in chiral Tellurium were investigated in detail by Tsirkin et al. [78] using first-principles methods.

At present, there exist no experimental methods to measure directly the vectorial Berry curvature and to distinguish the enantiomers in this manner. Moreover, as changes in the sign of $\Omega_{n}$ already appear through the inversion of the structure in achiral systems without an inversion center, they cannot be used to decide whether a structure is chiral. Therefore, they must be distinguished using results from circular dichroism and in particular circular dichroism in the angular distribution of photoelectrons. However, the Berry curvature is a ground state property whereas photoelectron spectroscopy deals with excited states and the ground state is included only in an indirect way.

### 7.4. Circular Dichroism, Chirality, and Electronic Structure

In contrast to the band structure, the optical absorption depends on the handedness of a crystal and measures the transition between occupied and unoccupied states. Obviously, the transitions between different states are affected by at least one physical quantity that is not observed from the usual electronic structure representations. Indeed, the band structure does not contain information regarding the phase of the wave functions related to the states. Photoemission, that is emission of electrons excited by photons, is complementary to photoabsorption and also exhibits dichroic effects. Photoabsorption or angular integrated photoemission average over the momentum space. Angular resolved photoelectron spectroscopy (see [79,80] and references there) offers the advantage of being able to scan the momentum (*k*) space. Therefore, the circular dichroism in photoemission allows the investigation of the interrelation between structural chirality and electronic structure.

The circular dichroism (CD) in photo absorption is typically a small effect (order $10^{-3}$ and less) that is based on electric dipole–electric quadrupole (E1–E2) or electric dipole–magnetic dipole (E1–M1) interaction. In contrast, large effects (order 1) can be observed when investigating the circular dichroism in the angular distribution of the photoelectrons (CDAD), which already arise in pure electric dipole approximation (E1–E1 interaction).

The CDAD is essentially a non-magnetic effect appearing even in the absence of spin–orbit interaction. It was first proposed by Ritchie for chiral molecules in the gas phase, where the effect was small [81,82]. Later it was found by Cherepkov that large effects should appear already for achiral but oriented molecules when a handedness is enforced by the experiment [83]. In particular, photon incidence and molecular orientation, and direction of electron emission should not be coplanar. This effect was demonstrated first through the experiments conducted by Westphal, Schönhense et al. for CO adsorbed on Pt [84,85]. Feder [86] showed that the effect also appears in photoemission from solids, which was later subsequently observed in experiments as well. Further, Fecher et al. [87,88] investigated the role of the surface on the CDAD in emission from solids. A recent, extensive review of the group of Schönhense explained the present experimental possibilities for investigating the electronic structure of solids employing CDAD [89]. To date, the CDAD with its handedness enforced by the handedness of the experiment has been used to investigate achiral systems. However, it is also a powerful tool for investigating the electronic structure of chiral solids as shown below.

The impact of the symmetry on the CDAD was investigated by Cherepkov and co-workers for oriented achiral systems belonging to the point groups $C_{2v}$ [90], $C_{3v}$ [91], and $C_{4v}$ [92], and more general under consideration of the solid surface [93]. It was found that the CDAD vanishes when the quantization axis, photon spin, and electron emission are collinear. In addition, it also vanishes in the mirror planes of the experiment. These symmetry considerations indicate the manner in which to use CDAD for investigation of chiral systems. To avoid a handedness enforced by the geometry normal incidence should be used where the surface normal coincides at best with the high symmetry (chiral) axis of the crystal. The dichroic response to a change of the helicity of the circularly polarized photons can subsequently be observed in normal emission of the electrons.

**Figure 19:**
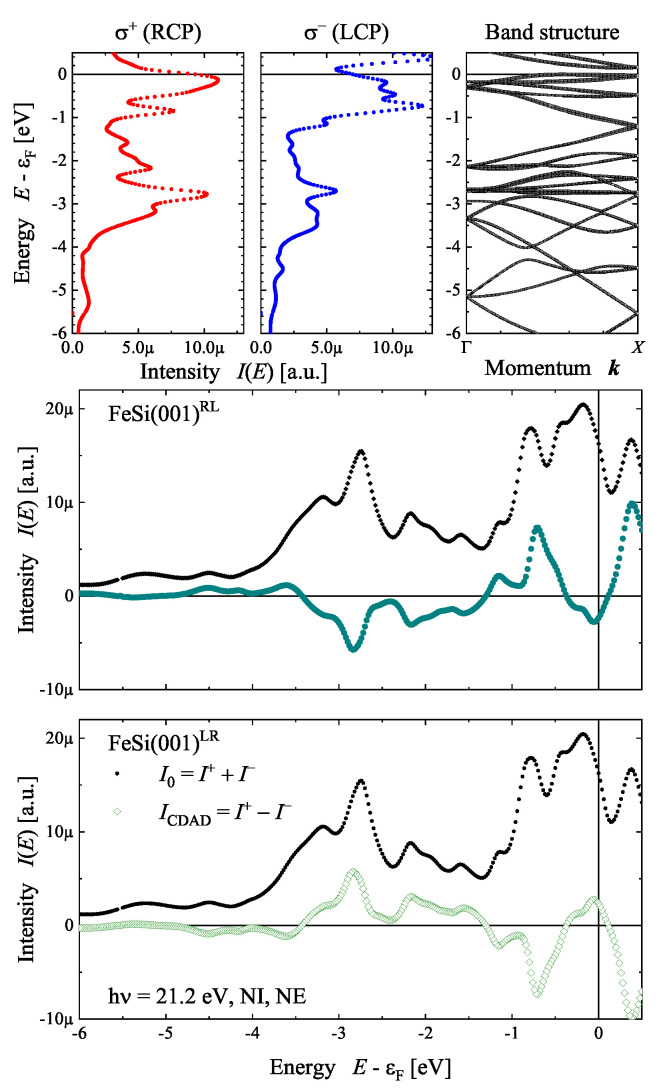
Polarization dependent photoelectron spectra and CDAD from FeSi(001). The polarization-dependent spectra are shown in comparison to the band structure along Δ for one of the enantiomers. The calculations are for normal incident photons of 21.2 eV energy and opposite helicity. For the enantiomorphous pair, the resulting circular dichroism is compared to the total intensity.

Figure 19 demonstrates the appearance of the CDAD from the FeSi(001) surface (see Figure 12) for the normal incidence–normal emission case using first principle calculations (Appendix A). The polarization-dependent spectra were calculated for a photon energy of hν=21.2 eV and were then compared to the band structure in the Δ-direction. The comparison allows us to determine which states contribute to the photoemission process. However, changing the photon polarization has a distinct effect, although the spectra are calculated in the direction of photon incidence. The largest positive dichroism appears at 3 eV below the Fermi energy ($\epsilon_{F}$), where it amounts to 42%, while the most negative value of −44% is observed at 0.7 eV below $\epsilon_{F}$. The normal emission spectra calculated for the RL enantiomer with $\sigma^{+}$ polarization cannot be distinguished from those for the LR enantiomer and $\sigma^{-}$ polarization. Consequently, the circular dichroism spectrum $I_{CDAD}=I^{+}-I^{-}$ has an opposite sign, whereas the bare intensity spectra with $I_{0}=I^{+}+I^{-}$ are the same. Further information regarding the handedness of the crystal is included in the angular distribution as will be demonstrated next.

The missing dichroic response in mirror planes of the achiral system enables the investigation of the handedness of chiral crystals. Using normal incidence along a high symmetry surface normal, the angular distribution of the electrons in the half-space above the surface reflects the symmetry of the crystal by the CDAD. An example is provided using VSi${}_{2}$ crystallizing with the chiral space groups 180 or 181.

**Figure 20:**
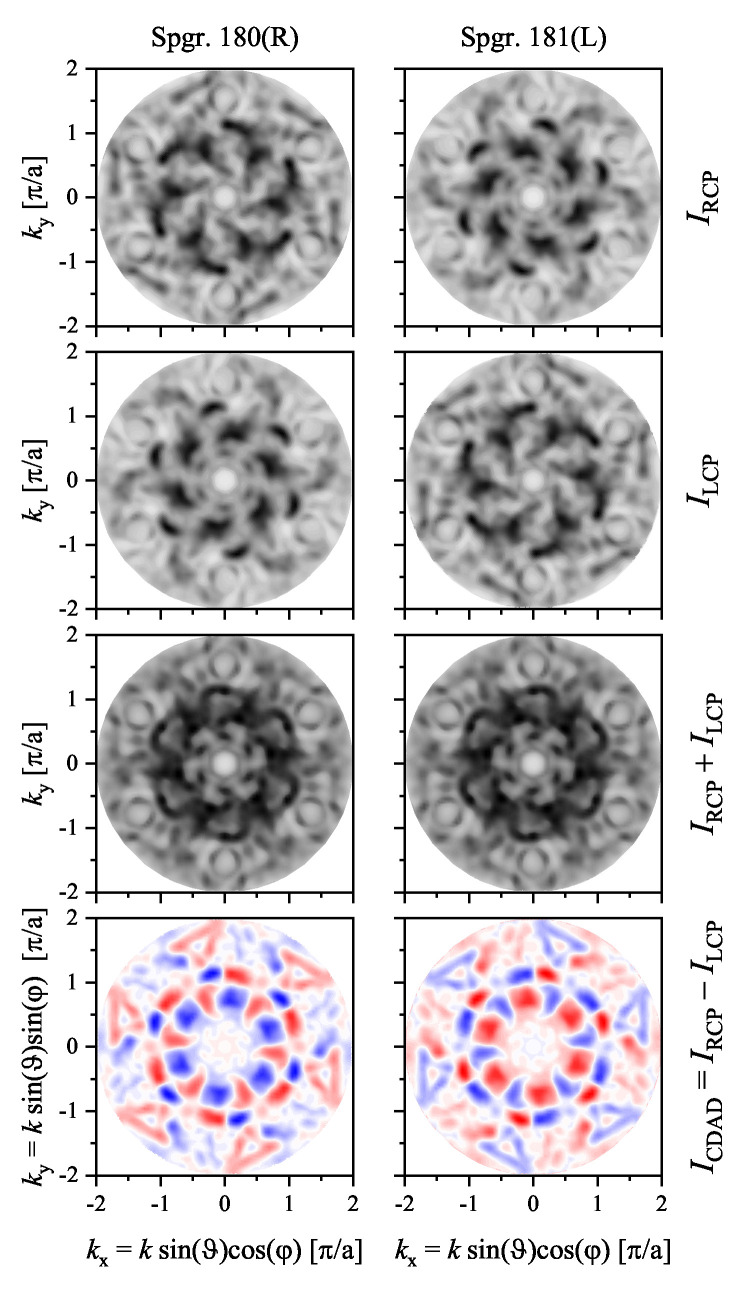
CDAD from VSi${}_{2}$(0001). The intensity distributions for excitation by circularly polarized photons of opposite helicity with normal incidence (that is along the (0001) surface normal) are shown. The intensities and circular dichroism (CDAD) from crystals belonging to the two space groups with opposite handedness are compared.

The circular dichroism in the angular distribution of the photoelectrons emitted from the hexagonal VSi${}_{2}$(0001) surface (see Figure 10 and Figure 14) is illustrated in Figure 20. It was chosen because it is metallic and thus has states crossing the Fermi energy. The calculations were performed for the normal incidence of the photons with an energy of hν=21.2 eV. Further, the energy of the photoelectrons was set to the Fermi energy and thus reflected a part of the Fermi surface. Consequently, the changes in the intensity distributions between the two enantiomers with changing photon polarization are striking and result in a memorable CDAD. The intensity distributions and the dichroism clearly reflect the absence of mirror planes and the rotational structure with opposite handedness. The maximum dichroism was ±53% with respect to the intensity maxima. Moreover, an integration of the angular distribution $I^{CDAD}\left(k_{x},k_{y}\right)$ shown in Figure 20 results in a circular dichroism of 1.86% for the right-handed ($P\;6_{2}22$, 180) surface and −1.86% for the left-handed ($P\;6_{4}22$, 181) one. Thus, the sign of the circular dichroism is opposite for the crystals with opposite handedness.

These two examples, one for a chiral structure and another for a chiral space group, demonstrate the manner in which angular resolved photoelectron spectroscopy can be used to investigate the electronic structure of chiral solids when using circular dichroism. The method is particularly interesting for metals that are not transparent for photons with optical wavelengths (infrared to visible light). Owing to its ability to scan the *k*-space it carries much more information compared to photoabsorption methods, including for example the natural X-ray circular dichroism [94] which also depends on the E1–E2 interference term. Moreover, the CDAD method is also suitable for investigating chiral surfaces or chiral molecules adsorbed at chiral or achiral surfaces. Recently, the relations between optical rotation, circular dichroism, and the chirality of light have been reviewed by A. Liniger et al [95]. The use of super-chiral and optimal chiral light will allow different pathways to examine chiral solids making use of novel dichroic effects.

## 8. Discussion, Summary, and Conclusions

The study presented an overview of chiral structures and chiral space groups, distinguishing structures from space groups. Chiral space groups are related to special but not neutral screw rotations as symmetry elements and always appear in enantiomorphous pairs. Two measures of chirality were introduced to quantify and distinguish chiral structures: Hausdorff distance and the continuous chirality measure. Examples of these characteristic quantities were reported for selected crystal structures. The discussion of the chirality measures revealed that the transition from achiral to chiral structures (and vice versa) is smooth and can be forced by very small displacements of the atoms forming the crystal structure. Further, the chirality was discussed for bulk solids and their surfaces where the latter is vital to catalysis and photoelectron spectroscopy. In addition, the chiral surfaces and the surfaces of chiral crystals were distinguished through several examples. For the electronic structure, it was found that its basic characteristic quantities, the band structure, and density of states, were affected by the same crystal parameters as the chirality. However, these quantities are independent of the handedness, implying that they stay the same when the handedness of the crystal structure is changed through mirror operations or inversion. Further, the Berry curvature depends on the handedness but is not proof of chirality because it reflects the inversion of a structure also in achiral crystals. Finally, it was proposed that the circular dichroism in the angular distribution of photoelectrons allows the distinguishing of the handedness of chiral solids and their surfaces. The chiral response of the photoelectron distribution excited by circularly polarized photons of opposite helicity was demonstrated through two examples. Consequently, the appearance of handedness of the experiment, which also produces dichroism, was avoided when using high symmetry, collinear configurations.

In the presented work, four main aspects were dealt with and have been concretized using various materials as examples:

(I) chirality, chirality measure, chirality sense, handedness, and helicity;
(II) chiral structures and chiral crystallographic space groups;
(III) chirality in two and three dimensions;
(IV) material properties depending on chirality measures or chirality sense.

In particular, it was shown that the 22 chiral space groups result in handed structures but not the 43 achiral Sohncke groups. Nevertheless, the chiral structures described by those achiral type III space groups can have opposite chirality senses, resulting in enantiomorphous pairs. Further, achiral bulk materials may have chiral surfaces. Chirality measures have been used to quantify chirality and to compare different chiral compounds with the same structure type. So far, chirality measures do not distinguish the chirality sense. It is also not seen in the standard quantities of the electronic structure of solids that are the density of states and band structure, even though chirality is definitely present in the charge density distribution through the crystal structure. Finally, it was demonstrated that circular dichroism in photoelectron spectroscopy is able to determine the chirality of the electronic structure.

## Figures and Tables

**Table 1:** Structure–symmetry–property relations in 7 non-centrosymmetric classes composed from the 21 non-centrosymmetric Laue classes.

		Property			
No.	Laue Class	E	PY	O	PI
**1**	**1, 2, 3, 4, 6**	⊠	⊠	⊠	⊠
**2**	**222, 32, 422, 622, 23**	⊠		⊠	⊠
**3**	**432**	⊠		⊠	
4	m,mm2		⊠	⊠	⊠
5	3m,4mm,6mm		⊠		⊠
6	$\bar{4},\bar{4}2m$			⊠	⊠
7	$\bar{6},\bar{6}2m,\bar{4}3m$				⊠

**E** = enantiomorphism (chirality), **PY** = polar (pyroelectric, ferroelectric), **O** = optical active, and **PI** = piezoelectric and nonlinear optics.

**Table 2:** The Sohncke groups: 65 space groups for chiral structures.

Crystal System	Laue Class	Point Group	Hermann-Mauguin Symbol	Space Group Number
Triclinic	1	$C_{1}$	P1	1
Monoclinic	2	$C_{2}$	P121, $P\;12_{1}1$, C121	3–5
Orthorhombic	222	$D_{2}$	P222, $P\;222_{1}$, $P\;2_{1}2_{1}2$, $P\;2_{1}2_{1}2_{1}$,	16–…
			$C\;222_{1}$, C222, F222, I222, $I\;2_{1}2_{1}2_{1}$	…–24
Tetragonal	4	$C_{4}$	P4, $P\;4_{1}$, $P\;4_{2}$, $P\;4_{3}$, I4, $I\;4_{1}$	75–80
	422	$D_{4}$	P422, $P\;42_{1}2$, $P\;4_{1}22$, $P\;4_{1}2_{1}2$, $P\;4_{2}22$, $P\;4_{2}2_{1}2$,	89–…
			$P\;4_{3}22$, $P\;4_{3}2_{1}2$, I422, $I\;4_{1}22$	…–98
Trigonal	3	$C_{3}$	P3, $P\;3_{1}$, $P\;3_{2}$, R3	143–146
	32	$D_{3}$	P312, P321, $P\;3_{1}12$, $P\;3_{1}21$, $P\;3_{2}12$, $P\;3_{2}21$, R32	149–155
Hexagonal	6	$C_{6}$	P6, $P\;6_{1}$, $P\;6_{5}$, $P\;6_{2}$, $P\;6_{4}$, $P\;6_{3}$	168–173
	622	$D_{6}$	P622, $P\;6_{1}22$, $P\;6_{5}22$, $P\;6_{2}22$, $P\;6_{4}22$, $P\;6_{3}22$	177–182
Cubic	23	*T*	P23, F23, I23, $P\;2_{1}3$, $I\;2_{1}3$	195–199
	432	*O*	$P\;4_{3}2$, $P\;4_{2}32$, F432, $F\;4_{1}32$, I432,	207–…
			$P\;4_{3}32$, $P\;4_{1}32$, $I\;4_{1}32$	… –214

**Table 3:** The 22 chiral space groups of class II in 11 enantiomorphous pairs (columns as in Table 2).

Tetragonal	4	$C_{4}$	$P\;4_{1}$, $P\;4_{3}$	(76,78)
	422	$D_{4}$	$P\;4_{1}22$, $P\;4_{3}22$	(91,95)
			$P\;4_{1}2_{1}2$, $P\;4_{3}2_{1}2$	(92,96)
Trigonal	3	$C_{3}$	$P\;3_{1}$, $P\;3_{2}$	(144,145)
	32	$D_{3}$	$P\;3_{1}12$, $P\;3_{2}12$	(151,153)
			$P\;3_{1}21$, $P\;3_{2}21$	(152,154)
Hexagonal	6	$C_{6}$	$P\;6_{1}$, $P\;6_{5}$	(169,170)
			$P\;6_{2}$, $P\;6_{4}$	(171,172)
	622	$D_{6}$	$P\;6_{1}22$, $P\;6_{5}22$	(178,179)
			$P\;6_{2}22$, $P\;6_{4}22$	(180,181)
Cubic	432	*O*	$P\;4_{3}32$, $P\;4_{1}32$	(212,213)

**Table 4:** Chiral and polar axes of the Sohncke groups. *c* is assumed to be a unique axis for the monoclinic system. The rhombohedral axes are assumed for trigonal systems (e.g., 32), for hexagonal setting Laue class 321 must be distinguished from 312. Single axes are denoted by [], groups of axes are denoted by <>.

Crystal System	Laue Class	Point Group	Chiral Axes	Polar Axes
Monoclinic	2	$C_{2}$	[001]	[001]
Orthorhombic	222	$D_{2}$	[001], [100], [010]	None
Tetragonal	4	$C_{4}$	[001]	[001]
	422	$D_{4}$	[001], [100], [010],	None
			[110], [$\bar{1}10$]	
Trigonal	3	$C_{3}$	[001]	[001]
	32	$D_{3}$	[111], [$1\bar{1}0$],	[$1\bar{1}0$],
			[$01\bar{1}$], [$\bar{1}01$]	[$01\bar{1}$], [$\bar{1}01$]
Hexagonal	6	$C_{6}$	[001]	[001]
	622	$D_{6}$	[001], [100], [010],	None
			[$\bar{1}10$], [$\bar{1}\bar{1}0$],	
			[210], [120]	
Cubic	23	*T*	$\langle 111\rangle$, $\langle 100\rangle$	$\langle 111\rangle$
	432	*O*	$\langle 111\rangle$, $\langle 100\rangle$, $\langle 110\rangle$	None

**Table 5:** Plane lattices and space groups. The 5 groups without mirror operations, that are hosts for chiral objects, are marked in bold. The numbering of the groups is according to Ref. [22].

Bravais Lattice	Point Group	Plane Group	Number
Oblique	$C_{1}$	p1	1
	$C_{2}$	p2	2
Rectangular	$C_{s}$	pm,pg	3, 4
	$C_{2v}$	p2mm,p2mg,p2gg	6, 7, 8
Rhombic	$C_{s}$	cm	5
	$C_{2v}$	c2mm	9
Square	$C_{4}$	p4	10
	$C_{4v}$	p4mm,p4gm	11, 12
Hexagonal	$C_{3}$	p3	13
	$C_{6}$	p6	16
	$C_{3v}$	p3m1,p31m	14, 15
	$C_{6v}$	p6mm	17

**Table 6:** Atom positions in cubic, achiral and chiral structures related to space group $P\;2_{1}3$. Tabulated are the positions for u≤1/8. Others may be found using a shift vector (n4,n4,n4) (n=1,2,3) and the equivalence of positions with −u and 1−u. The minimum distance of the positions between $P\;2_{1}3$ and $F\;m\bar{3}m$ is in all four cases $u\sqrt{3}$.

$F\;m\bar{3}m$	$P\;2_{1}3$	$P\;4_{3}32$
(0,0,0)	(u,u,u)	(18,18,18)
(12,12,0)	(12+u,12−u,−u)	(58,38,−18)
(12,0,12)	(12−u,−u,12+u)	(38,−18,58)
(0,12,12)	(−u,12+u,12−u)	(−18,58,38)

**Table 7:** Chirality of WO$Z_{4}$ compounds (Z= Br, Cl). Given are the unnormalized Hausdorff distances, *h*, and continuous chirality measures, *s*, with respect to different possible achiral structures.

Achiral Group	Polar	WOBr${}_{4}$		WOCl${}_{4}$	
		h	s	h	s
I4/m	N	0.049	0.0650	0.045	0.0642
I4/mm	Y	0.049	0.0654	0.045	0.0645
I4/mmm	N	0.049	0.0818	0.045	0.0800

**Table 8:** Chirality of compounds with C40 structure. Given are the lattice (a,c) and position (*u*) parameters, as well as the continuous chirality measures ($S^{2}$). Lattice parameters are from the Pearson database [20].

	*a* [Å]	*c* [Å]	*u*	$S^{2}$
CrSi${}_{2}$	4.4283	6.3680	0.1658	0.2706
MoSi${}_{2}$	4.6220	6.6460	0.1642	0.2702
NbSi${}_{2}$	4.7974	6.5923	0.1593	0.2690
TaSi${}_{2}$	4.7839	6.5700	0.1590	0.2689
VSi${}_{2}$	4.5726	6.3744	0.1626	0.2698
WSi${}_{2}$	4.6180	6.6740	0.1640	0.2702
NbGe${}_{2}$	4.9670	6.7830	0.1631	0.2699
TaGe${}_{2}$	4.9380	6.7300	0.1640	0.2702
WAl${}_{2}$	4.7422	6.6057	0.1618	0.2696

**Table 9:** Symmetry of special projections of the chiral space groups. Chiral projections are marked by printing their plane groups (pi, i=1,3,4,6) in bold. The cubic, achiral Sohncke group 198 is given for comparison.

cubic	[001]	[110]	[111]
212,213	p4gm	p2gm	p3m
198	p2gg	pg	p3
hexagonal	[0001]	[11$\bar{2}$0]	
180,181	p6mm	p2mm	
178,179	p6mm	p2gm	
171,172	p6	pm	
169,170	p6	pg	
trigonal	[001]	[100]	[210]
152,154	p3m	p2	pm
151,153	p3m	pm	p2
144,145	p3	p1	p1
tetragonal	[001]	[100]	[110]
92,96	p4gm	p2gg	p2gm
91,95	p4mm	p2gm	p2gm
76,78	p4	pg	pg

## Data Availability

Data are available from the authors on reasonable request.

## Appendix A. First Principles Calculations

In the main part, certain calculated band structures were shown. The reported electronic structures were investigated with ab initio calculations using the full potential linearized augmented plane wave method as implemented in Wien2k [96]. The exchange–correlation functional was considered within the generalized gradient approximation in the parameterization of Perdew et al. [97]. For integration, 16,000 *k*-points of the full Brillouin zone were used for cubic and 9000 *k*-points for hexagonal structures. The final numbers of *k*-points were reduced to the irreducible wedges of the Brillouin zones and thus depend on the symmetry. Further, the energy convergence criterion was set to $10^{-5}$ Ry, and simultaneously the criterion for charge convergence to $10^{-3}e^{-}$. In addition, a force criterion of 0.2 mRy/$a_{B}$ ($a_{B}$ = Bohr’s radius) was used during the optimization of the free *u* parameters and full structure optimizations. Wannerisation was performed using Wien2Wannier [98] and the Wannier functions have been analyzed with Wannier90 [99]. Photoelectron spectra were calculated with the spin-polarized full relativistic Munich SPRKKR code [100,101] with modifications based on the full relativistic slab code RSLAB of Braun [102]. Both codes are based on Korringa–Kohn–Rostoker (KKR) type multiple scattering methods and use four-component Dirac-type wave functions. Here, spin–orbit interaction is an intrinsic property of the Dirac equation and is not introduced as a perturbation.

## Appendix B. Relation Between Chiral Structures, Symmorphic Space Groups, and Topological Spaces

The only allowed symmetry operations in symmorphic space groups are pure rotations, pure reflections, and roto-reflections, of which the latter two result in achiral structures. Combining the 32 point groups with the allowed translational lattice types, 73 out of the 225 space groups are found to be symmorphic. The symmorphic groups are listed in Table A1. Further, 23 out of these groups contain only proper rotations and thus, also belong to the set of Sohncke groups. However, none of these space groups belonging simultaneously to both sets is chiral by itself although they describe chiral structures, implying that all symmorphic space groups are achiral even though certain structures belonging to those groups might be chiral. In other words, all chiral space groups are asymmorphic.

A total of 35 out of the 65 Sohncke groups have orbifolds belonging to $S^{3}$ (3 spheres) topological space. Only 13 out of these are symmorphic. They are marked in Table A1. Five pairs of the non-symmorphic Sohncke groups belonging to $S^{3}$ are chiral, namely $P\;4_{1,3}22$, $P\;3_{1,2}12$, $P\;6_{1,5}22$, $P\;6_{2,4}22$, and $P\;4_{1,3}32$.

**Table A1:** The 23 symmorphic Sohnke groups. Printed in bold are those groups belonging to $S^{3}$ (3 spheres) topological space.

Crystal System	Space Group
Triclinic	P1
Monoclinic	P2, C2
Orthorhombic	P222, C222, I222, F222
Tetragonal	P4, P422, I4, I422
Trigonal	P3, P312, P321, R3, R32
Hexagonal	P6, P622
Cubic	P23, I23, I432, F23, F432
